# Systems protobiology: origin of life in lipid catalytic networks

**DOI:** 10.1098/rsif.2018.0159

**Published:** 2018-07-25

**Authors:** Doron Lancet, Raphael Zidovetzki, Omer Markovitch

**Affiliations:** 1 Department of Molecular Genetics, Weizmann Institute of Science, Rehovot 76100, Israel; 2 Department of Molecular, Cell and Systems Biology, University of California, Riverside, CA 92521, USA; 3 Origins Center, Center for Systems Chemistry, Stratingh Institute for Chemistry, University of Groningen, Groningen, the Netherlands; 4 Blue Marble Space Institute of Science, Seattle, WA, USA

**Keywords:** origin of life, prebiotic evolution, reflexively autocatalytic sets, composome networks, metabolism first, pre-RNA world

## Abstract

Life is that which replicates and evolves, but there is no consensus on how life emerged. We advocate a systems protobiology view, whereby the first replicators were assemblies of spontaneously accreting, heterogeneous and mostly non-canonical amphiphiles. This view is substantiated by rigorous chemical kinetics simulations of the graded autocatalysis replication domain (GARD) model, based on the notion that the replication or reproduction of compositional information predated that of sequence information. GARD reveals the emergence of privileged non-equilibrium assemblies (composomes), which portray catalysis-based homeostatic (concentration-preserving) growth. Such a process, along with occasional assembly fission, embodies cell-like reproduction. GARD pre-RNA evolution is evidenced in the selection of different composomes within a sparse fitness landscape, in response to environmental chemical changes. These observations refute claims that GARD assemblies (or other mutually catalytic networks in the metabolism first scenario) cannot evolve. Composomes represent both a genotype and a selectable phenotype, anteceding present-day biology in which the two are mostly separated. Detailed GARD analyses show attractor-like transitions from random assemblies to self-organized composomes, with negative entropy change, thus establishing composomes as dissipative systems—hallmarks of life. We show a preliminary new version of our model, metabolic GARD (M-GARD), in which lipid covalent modifications are orchestrated by non-enzymatic lipid catalysts, themselves compositionally reproduced. M-GARD fills the gap of the lack of true metabolism in basic GARD, and is rewardingly supported by a published experimental instance of a lipid-based mutually catalytic network. Anticipating near-future far-reaching progress of molecular dynamics, M-GARD is slated to quantitatively depict elaborate protocells, with orchestrated reproduction of both lipid bilayer and lumenal content. Finally, a GARD analysis in a whole-planet context offers the potential for estimating the probability of life's emergence. The invigorated GARD scrutiny presented in this review enhances the validity of autocatalytic sets as a *bona fide* early evolution scenario and provides essential infrastructure for a paradigm shift towards a systems protobiology view of life's origin.

## 1. Mutually catalytic networks

NASA's widely accepted definition of minimal life asserts that ‘Life is a self-sustaining chemical system capable of Darwinian evolution’ [1–3, p. 217]. Two schools of thought attempt to instil chemical realism into this definition. A majority opinion (RNA first) contends that the first self-replicating and evolving entities were informational biopolymers [4–6]. An alternative view (affiliated with ‘metabolism first’) claims that life began with mutually catalytic networks of smaller molecules, endowed with self-replication^1^ and evolution capabilities [7]. This dichotomy has been lucidly stated as follows: ‘One mechanism, based on quasispecies … has self-replicating entities as its components. Another proposed mechanism starts from simpler components that are not individually self-replicating but can collectively form an autocatalytic set’ [9, p. 5684]. Likewise, the catalytic network view is described as ‘a protocell system consisting of a large number of molecule species that catalyze each other … (which) can establish recursive production’ [10, p. 782]. What seems to be shared by both schools is that ‘The formation of a self-sustaining autocatalytic chemical network is a necessary but not sufficient condition for the origin of life’ [11, p. 3085]. This review strives to carefully assess the validity of the autocatalytic set school of thought, and seek evidence for its legitimacy as a *bona fide* scenario for life's origin.

Despite the strong popularity of the ‘RNA-first’ view, the alternative has gained considerable foothold. This is exemplified by statements such as: ‘Many scientists believe life began with the spontaneous formation of (an RNA) replicator … . A more likely alternative for the origin of life is one in which a collection of small organic molecules multiply their numbers through catalyzed reaction cycles, driven by a flow of available free energy’ [12, p. 105]; ‘Metabolism first scenarios are … gaining acceptance as both more plausible and potentially more predictive’ [13, p. 13168] and ‘In contrast to the sophisticated high-fidelity nucleic acid-based inheritance, … I hypothesize a lower fidelity predecessor where a simpler, less-exact stepwise process gave rise to the first hereditary information system’ [14, p. 294]. A succinct statement of this scenario, along with simulation evidence, is found in a paper entitled ‘Complex autocatalysis in simple chemistries’ [15].

It is interesting that the disagreement has begun quite early, between the noted geneticist Hermann Muller and the origin of life pioneer Alexander Oparin, as described [16, p. 373]: ‘Whereas for Oparin life was the outcome of the step-wise slow process of precellular evolution in which membrane-bounded polymolecular systems played a key role, Muller argued that life started with the appearance of the first nucleic-acid (DNA) molecule in the primitive oceans’. This dispute has definitely not been put to rest. Some of the best advocacies for Oparin's stand have been put forth by Dyson in his book ‘Origins of Life’ [8], by Kauffman [17, p. 1], proclaiming that ‘reflexively autocatalytic sets of peptides … may be an … inevitable collective property of any sufficiently complex set’ and by Shapiro's writing [18, p. 173] that ‘(while) the formation of the first replicator through a very improbable event cannot be excluded … greater attention should be given to metabolism first theories which avoid this difficulty’.

The experimental exploration of mutually catalytic networks has been considered challenging [19]. Recently, there have been rising experimental interest in network collective behaviour in the origins of life context [20–24]. However, the classical autocatalytic set models [8,17] have largely eluded experimentation. One possible reason for this paucity relates to the adamant conceptual doubts regarding the capacity of mutually catalytic networks (as opposed to RNA systems) to support self-replication/reproduction and Darwinian evolution [25–27]. In an attempt to alleviate these doubts, we examine herein the recent progress in exploring mutually catalytic networks via simulateable quantitative chemical kinetics models, with focus on the example of our graded autocatalysis replication domain (GARD) model [28].

It is legitimate to point out the paucity of experimental evidence for mutually catalytic networks. But it is noteworthy that every extant living cell constitutes experimental verification for this concept. A cell is a highly complex web of mutually interacting chemical components, which include not only metabolites and membrane-forming lipids, but also informational and functional biopolymers—DNA, RNA and proteins. Such biopolymers indisputably fulfil a central role in cellular information transfer and decoding, thus being the crux of what present life is. But in the final account, informational biopolymers constitute part of metabolism, with monomer-synthesis, monomer activation and catalysis-dependent controlled polymerization. It is thus obvious that a cell is capable of self-sustaining and self-replicating its entire content via an intricate mutual catalysis web (cf. [29]). By contrast, present-day cells cannot exemplify self-replicating informational polymer, because no individual cellular molecule can directly instruct its own formation when in isolation. The key open question is whether a much simpler assemblage of molecules, devoid of biopolymers, may still conform to NASA's definition of life.

In present-day life, the cell cycle begins in the G_1_ phase whereby as the cell grows in volume, the entire non-DNA cell contents are catalytically duplicated, so as to keep the concentrations unchanged for all intracellular components (metabolites, lipids, proteins, RNAs). This is followed by the replication of DNA in the S phase, and ends with cell division in M phase [30]. In simpler molecular assemblies, there may be no DNA to replicate, and physical fission constitutes a bare-bone simile of the M phase. What needs to be pondered is how primitive catalytic assemblies may recapitulate the G_1_ phase—growth with concentration preservation, known as *homeostatic growth*. In such growth mode, the *ratios* among the quantities of all molecule types remain largely unchanged. The key player in the G_1_ phase of nowadays cells is the broadly defined metabolism, which includes transcription, translation and biosynthesis. Cellular metabolism thus has to be viewed not as just providing all the needed cellular molecules, but also as doing so in an exquisitely orchestrated fashion, which keeps all the inter-compound ratios unchanged upon volume doubling [31].

Thus, we should ask whether any published instance of primordial mutually catalytic networks (or metabolism) can show the phenomenon of concentration homeostasis. This likely imposes stringent quantitative constraints on the way by which the catalytic network is constructed. This is insightfully stated by Sharov [32, p. 11], in the context of a model for primordial life without nucleic acids: ‘Not every autocatalytic set … can support self-reproduction. Self-reproduction is possible only in autocatalytic sets with specific stoichiometry constraints, where a sequence of internal reactions can increase the number of all molecular species within the set’ (see elaboration in §3).

Network models such as autopoiesis [33,34], which provide only qualitative definitions without explicit kinetics are inadequate for homeostasis-related scrutiny. The Chemoton model [35] consists of three stoichiometrically coupled autocatalytic cycles: metabolism, template replication and membrane, with simulateable internal feedback that couples membrane and content growth [36]. Yet, Chemoton analyses have not so far quantitatively address network homeostasis.

A pioneering elaboration of a mutually catalytic set is Kauffman's reflexively autocatalytic set formalism [37,38], further expounded by Hordijk *et al.* [39]. The basic model ascribes a constant probability *p* to catalytic events in an entire molecular network, i.e. regarding catalysis as a binary phenomenon (yes or no catalysis). It is then shown that when a system reaches a sufficiently large diversity of molecule types, autocatalytic sets would appear spontaneously. These will have the property of ‘catalytic closure’, whereby the formation of every molecule is endogenously catalysed. It is then argued that a catalytically closed network is endowed with self-reproduction capacities, but homeostatic growth is not directly addressed (see §5.1). The same is true for several more recent studies of mutual catalysis-based network systems, exemplified by a paper on folding hetero-oligomers [40]. This describes how certain chains of mixed hydrophilic/hydrophobic monomers fold, then serve as mutual catalysts for the elongation of others, but the analyses provided do not account for homeostatic growth.

## 2. Chemical opportunism

There is another difference of opinions between two camps in the study of life's origin, involving the chemistry that might have prevailed at the early stages of life. The first opinion is that even early in life's emergence the chemistry was identical or very similar to that found within living cells today. This notion has instructed hundreds of studies seeking abiotic synthesis paths for many small and large present life compounds, proposing that they will somehow come together to form the first living entity [41,42]. We note that many of these experiments were conducted by what has been described as ‘school chemistry’ [43], i.e. ‘using modern apparatus and purified reagents’ [12, p. 105]. This approach has been criticized for ‘seldom considering the likelihood … (of synthesis) in the context of the early Earth’ [12, p. 106].

Of note, when considering the graded and ever-changing way in which evolution transpires, there is no compelling *a priori* reason to assume that life began with present-day life-characterizing molecules. This is echoed in the statement [14, p. 293]: ‘A central concept applied so far in origin of life research is based on the premise that if synthesis of a compound under prebiotic conditions occurred, then it is feasible to have played a role in prebiotic evolution. Considering that the timescale of the above events may be more than a billion years, any system that propagates molecular and catalytic diversity … could explain abiotic synthesis of many of the molecules of life’.

The second viewpoint asserts that life may have begun with chemistries very different from those found in contemporary organisms. This dissenting approach is stated as follows [44, p. 440]: ‘It is unlikely that under prebiotic conditions the complex and sophisticated biomacromolecules commonplace in modern biochemistry would have existed. Thus, research into the origin of life is intimately associated with the search for plausible systems that are much simpler than those we see today’. Similarly, it is pointed out that ‘Biochemistry, as we know it, occupies a minute volume of the possible organic “chemical space”. As the majority of abiotic syntheses appear to make a large set of compounds not found in biochemistry, as well as an incomplete subset of those that are, it is possible that life began with a significantly different set of components' [45, p. 1].

The latter point of view carries the meaning that the early steps towards life were ‘opportunistic’, whereby it was much less important which specific compounds were involved, as long as they had the right chemical characteristics, such as catalysis, energy mediation or membrane formation. This implies also that a very large number of different molecular configurations could have been involved in such early life progressions. That is the situation invoked in Oparin's ‘primordial soup’ [46], in Dyson's ‘Garbage bag’ scenario [8] and in Lazcano's rendering that ‘the prebiotic soup must have been a bewildering organic chemical wonderland’ [25, p. 73]. If indeed life began with a chemistry much different from that of present-day cells, it is intriguing to explore to what degree living cells today are palimpsests, showing some hints of much earlier chemistry.

Early replicators of the mutually catalytic set type are high on the opportunism scale, as they do not usually pose strong constraints on the chemical configurations involved. In the GARD/Lipid World model, presented in the following sections, the members of the mutually catalytic set are assumed to be amphiphiles, without stating any further limitations, hence may be referred to as having high opportunism. In fact, a very large initial molecular repertoire is a necessary condition for the GARD model to operate [47]. In general, a high level of opportunism enhances the probability ascribed to a life's origin scenario. Thus, a ‘choosy’ RNA-based model, requiring strictly specified compounds to be sampled out of a highly diverse repertoire, is much less probable as life's first step than opportunistic mutually catalytic sets.

Additional support to high opportunism scenarios has been voiced [48, p. 3]: ‘Ubiquity is a principle that favors origin scenarios taking place within common or widespread environmental conditions over highly specialized or rare environments. Miller–Urey style amino acid synthesis can only take place in reducing atmospheres, and once it was realized that those conditions were unlikely [49] … , commitment to the ubiquity principle would seem to suggest abandoning Miller–Urey approaches'.

## 3. Compositional homeostasis

To fathom how opportunistic scenarios can lead to ensemble reproduction, a more detailed view of catalytic networks is needed. As said, homeostatic growth of a molecular assembly happens when the ratios among the concentrations of its components remains unchanged along a growth trajectory. In other words, as the assembly grows in volume, the counts of all its molecule types increases in proportion to their original values, so as to keep all internal molar fractions unchanged. In a more formal description, for *N*_G_ types of molecule, *A*_1_,*A*_2_, … *A_i_*, … ,*A*_NG_, an assembly's composition is fully described by an *N*_G_-dimensional compositional vector **n** = (*n*_1_,*n*_2_, … *n_i_*, … ,*n*_NG_), where *n_i_* are the counts of the molecules *A_i_*. As the assembly grows, if the length of the vector **n** increases while its direction remains unchanged, then the growth is homeostatic, stemming from the kinetic intricacies of the catalytic network (see below). This process represents a prerequisite for copying of compositional information, an alternative to the copying of sequence information by templating biopolymers (see §5.2). Such pursuit of compositional preservation has in parallel been described by Kaneko [50] and Baum [29].

**Figure 1.**
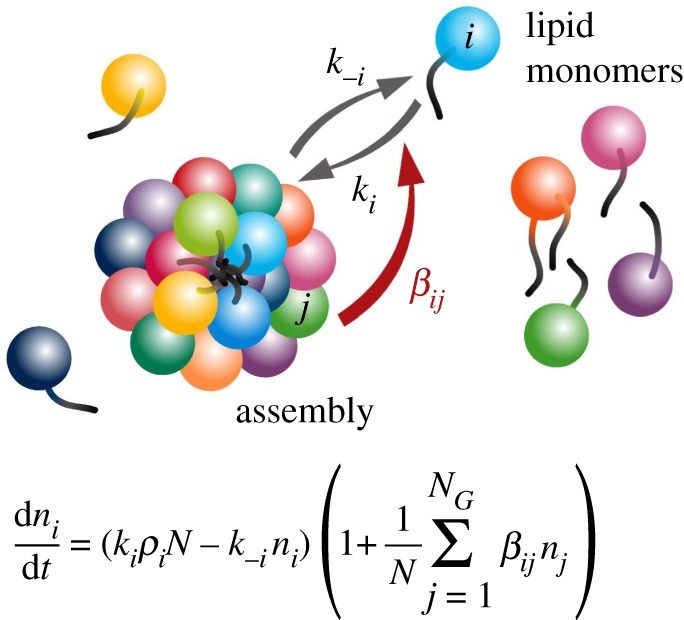
The graded autocatalysis replication domain (GARD) model is based on computer simulations of rigorous chemical behaviour. The model involves a stochastic chemistry simulation based on a set of differential equations as shown. The main reaction step is the entry and exit of an amphiphilic molecule *A_i_*, belonging to a repertoire of *N*_G_ amphiphile types (represented by different colours), between the environment and an assembly (in this figure exemplified by a small micelle). The variable *n_i_* is the count of *A_i_* molecules within the assembly, *N* = *Σn_i_*, the total count of all *N*_G_ species in the assembly, *k_i_* and *k*_−*i*_ are, respectively, the basal (spontaneous) forward and backward rate constants for *A_i_*, (black arrows), and *ρ_i_* is the external concentration of *A_i_*. A key aspect, crucial for reaching a kinetically controlled homeostatic growth of the assembly, is the dependence of the reaction rates on the current composition of the assembly. This dependence is controlled by a matrix *β*, whose elements *β_ij_* are the rate-enhancement values for internal compounds on the rate of the exchange reaction. The matrix element *β_ij_* signifies the rate-enhancement parameters for the catalysis exerted by the in-assembly species *A_j_* on the joining and leaving reactions of *A_i_* (red arrow). The matrix elements thus control the dynamics of the mutually catalytic network embodied in the GARD assembly, and its elements are drawn from a probability distribution generated through the RAD model (§4) [65].

**Figure 2.**
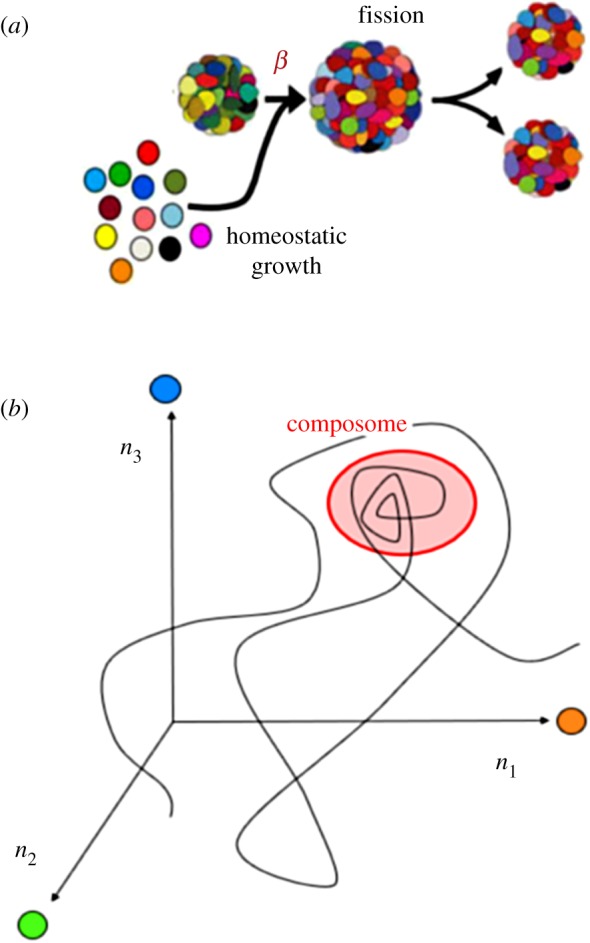
(*a*) The numerical solutions in the simulation of GARD dynamics show that for certain sets of amphiphile counts (composomes, see panel (*b*)) homeostatic growth is observed. This stems from molecular entry rates that are proportional to the molecular counts inside the assembly. Upon assembly growth, occasional assembly fission results in the generation of progeny. In the simulation, growth is modelled to occur with the total molecule count *N* increasing from *N* = *N*_MAX_/2 = *N*_MIN_ to *N* = *N*_MAX_. If the assembly is in a composome state and the condition *N*_MIN_ ≥ *N*_MOL_ is fulfilled, then fission will statistically generate two similar progeny, both also similar to the pre-growth assembly. Thus, the growth–fission process is equivalent to assembly replication or reproduction. (*b*) GARD provides a detailed molecular description of a walk in compositional space, shown here in a three-dimensional principal component diagram derived from a 100-dimensional compositional space. The trajectory covers many growth–fission events, in a simulation in which after each fission, one progeny assembly is discarded, so the ‘trace’ focuses on one assembly at any given time. The trajectory portrays the emergence of a compositional quasi-stationary state, termed composome, whereby the compositional vector (a point in compositional space) remains largely unchanged over several growth–split cycles. When in a composome state, an assembly preserves its composition by homeostatic growth. Importantly, the reproduction of a composome is an emergent phenomenon, stemming from the chemical kinetics equations that governs its dynamics (figure 1). This is in clear contrast to other scenarios, such as the quasi-species model, in which a modelled polynucleotide is *assumed* to have replication capacity. As GARD assemblies store information in the form of non-random molecular compositions (figure 4) and transfer this information to fission-generated progeny, their behaviour is defined as compositional replication/reproduction (or compositional inheritance).

Over the last 20 years, we have studied a specific case of mutually catalytic networks called GARD [28,47,51–64]. GARD is specified in explicit kinetic equations, amenable to computer simulations (figure 1) and explicitly assumes that the participating molecules are amphiphiles that spontaneously form discrete assemblies [55]. Our published analyses of the ensuing dynamic behaviour of GARD clearly reveal a capacity for homeostatic growth, which is shown to result in, and be a prerequisite for compositional inheritance [54,56]. In combination with random fission of the grown assembly, induced by, e.g. shear forces or thermodynamic instability [66,67], this entire process constitutes compositional self-reproduction. This property is portrayed only by certain assemblies, which happen to have the appropriate molecular composition, termed *composomes* (figure 2, see §5). In GARD, the rate of amphiphilic monomer incorporation is dictated kinetically by the current assembly composition (figure 1). Such a dependency is analogous to that invoked by Nowak [68] for prebiotic selection involving template-free elongation of polymers within compartments. In Nowak's model, there is influence of sequence motifs on the rate of incorporation of new monomers into growing polymers. The probable importance of network interactions and molecular compositions in early evolution is accentuated in the words of Lehman and co-workers: ‘The origins of life likely required the cooperation among a set of molecular species interacting in a network. If so, then the earliest modes of evolutionary change would have been governed by the manners and mechanisms by which networks change their compositions over time’ [24, p. 3206].

GARD is a bare-bone model, intended as a proof of concept, yet includes rigorous and accurately specified chemical features that make it all but an abstract, theoretical toy model [53]. GARD's genre is sometimes defined as artificial chemistry [15,69], as described in [70]. But in many respects it is a coarse-grain molecular dynamics model, strictly capturing the laws of physics and chemistry, and ripe for more extensive molecular dynamics simulations (see §14.1).

In a discerning review, Higgs [71, p. 225] makes a distinction among three stages in the origin of life, whereby ‘chemical evolution is an important stage on the pathway to life, between the stage of “just chemistry” and the stage of full biological evolution’. He defines his own model, as well as our own GARD model, as belonging to the chemical evolution stage, with replication and Darwinian evolution, but still ‘not quite constitute(ing) life’. One may ask what attributes are portrayed by biological evolution but not by chemical evolution. Higgs' answer includes: (i) selection by encoded function; (ii) evolutionary open-endedness, i.e. a capacity to access an entire fitness landscape, as opposed to just a local peak and (iii) encompassing ‘molecules that can only be produced by a replication process’.

Higgs decides ‘to put a boundary between non-life and life (at the) boundary between chemical evolution and biological evolution’. In contradistinction, we adhere to the strict NASA definition of life, calling GARD composomes ‘Life’. However, there is no true disagreement: Higgs states ‘…I agree that definitions are not an end in themselves, (but) I think that having clear definitions can actually help us to understand the processes involved in the origin of life’. Further, he pronounces that ‘The chemical evolution stage … is probably necessary to get true biological evolution going’. We fully agree that GARD is not full-fledged biology, and along with Higgs, as further detailed below, seek how as a chemical system that mutates, replicates and evolves, GARD can lead to biology.

## 4. Mutual catalysis matrices

In a generalized mutually catalytic network, the nodes correspond to the *N*_G_ molecule types and the (directed, weighted) edges are the mutual catalysis values. Such a network may thus be represented by an *N*_G_ × *N*_G_ square non-symmetric matrix (often called *β* in this review) whose positive elements *β_ij_* represent the network edges. In reality, such values are determined by the chemical nature of the substances involved. However, until such values can be inferred *ab initio* (§14.1), it is necessary to resort to one of several possible ways to populate this matrix while preserving a significant degree of realism.

In the original Kauffman model, the matrix elements have binary values (yes/no catalysis), with a constant appearance probability *p* for any of the reactions. Thus, considering *N*_G_ = 100 compounds with 10 000 mutual catalysis terms, in Kauffman's original definitions, if *p* = 0.02, 200 matrix entries will have a constant (usually unspecified) *β_ij_* > 0 and the rest of the elements will be *β_ij_* = 0. This in itself does not ensure that each of the 100 compounds will receive at least one catalytic influence (Kauffman's catalytic closure condition), but it has been demonstrated in an example of increasingly long peptides, that as *N*_G_ goes up, the probability of catalytic closure will approach 1 [72].

More generally, some models invoke different values for the matrix element *β_ij_* for each of the reaction, representing the idiosyncratic mutual catalysis exerted by molecule *A_i_* on molecule *A_j_*. A variegated matrix stands to reason in view of the potential diverse prebiotic chemistries. In this general case, one should assume that *β_ij_* are graded (weighted) positive non-zero values and that the matrix is, in general, non-symmetric. A somewhat unexpected result is that even with a low number of compound types (*N*_G_), with the above assumptions, every reaction receives some (but often very weak) catalysis. Under such circumstances, networks of any size will be catalytically closed. However, to conform to chemical realism, it is appropriate to consider a lower limit for discernible catalysis. Then, in a randomly defined set of compounds, only certain subsets may show catalytic closure [73,74].

Choosing *β_ij_* values at random is an acceptable first-tier strategy, particularly in the light of the currently limited knowledge of peptide or lipid catalysts. But from a chemical point of view, it is reasonable that in a large assortment of compound pairs some ranges of catalytic values will be more probable than others. Thus, one would guess that it is much more likely to encounter weak catalysis events than strong ones. One procedure aimed at quantifying this intuition is the use of bit string matching algorithms, representing polymers with a two-strong monomer repertoire. Bit string representations of molecular structure have often been applied to the more natural case of sequential oligomers, among others to model early evolution [75,76]. The gist of this approach is that the count of matched bits between two strings reflects the cumulative free energy of binding arising from numerous sub-site interactions. A similar concept has been applied to molecules with more general (non-sequence-based) configurations, including for simulating protein–protein interaction networks, such as the immune system (interactions of antibodies and antigens) [77] and ligand–receptor interactions in drug screening [78] and in the olfactory system [65].

**Figure 3.**
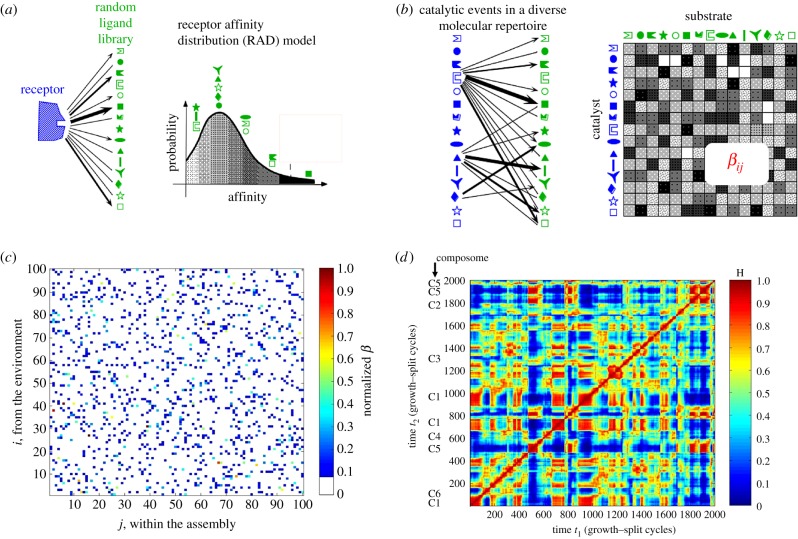
(*a*) The analogy of random chemistry principles between current experimental systems and early primordial scenarios. Nowadays, random library screening (e.g. with a phage display library) is used for searching a maximal affinity ligand (thick arrow, left) for receptors or antibodies. Statistical models for the affinity distribution that governs such selection (right) [65] allow one to quantitate this process, darker shades denoting higher affinity with the highest affinity ligand being at the far right end of the distribution, i.e. with a relatively low probability. (*b*) GARD assumes that a similar statistical distribution prevailed when both binders and ligands (or catalysts and substrates) were members of a randomly formed mixture of prebiotic small organic molecules (left), as embodied in the GARD model. The mutual interactions (shown in arrows of various thickness, signifying catalytic intensity) are documented in a matrix *β* (right), encompassing all the cross-catalytic and autocatalytic rate enhancements, with a similar darkness code as in *a*. Panels *a* and *b* are modified from [53]. (*c*) *β* matrix for a typical simulation. Colour code (shown on the bar) represents *β_ij_* values, normalized as described [61]. It is evident that strong diagonal elements, signifying autocatalysis, are statistically rare. The colour white (normalized *β_ij_* < 0.1) creates a graphical representation of cut-off as discussed in §5.1. This figure underlines the futility of attempting to functionally dissect a complex mutualistic grid by simpler terms of autocatalysis and dual catalysis [74]. Figure is modified from [61]. (*d*) Compositional correlation diagram of a GARD system, as described [54], with molecular repertoire size *N*_G_ = 100 and maximal assembly size *N*_MAX_ = 80. The drawing depicts a time correlation matrix, where both the ordinate and the abscissa represents the same timescale for the evolution of a particular GARD assembly for 2000 growth–split cycles. Each point in the two-dimensional graph is coloured (colour bar) by the normalized dot product *H* between the compositional vectors at times *t*_1_ and time *t*_2_, as described [61]. Near-diagonal red squares represent time periods of high compositional similarity across many (often several dozen) growth–split cycles, constituting composomes, marked Ci near the left axis. Off-diagonal colours allow one to infer the inter-composome similarity. Transitions from one composome to another, viewed along the diagonal, are estimated to occur within no more than 50 growth–split cycles. The simulation was conducted as described [54]. Figure is modified from [47].

Going one step further, one may acquire knowledge on the functional form of mathematical distribution that governs the mutual interaction values. This notion has been presented as seeking ‘a distribution of match strengths which reflect the energy of binding between catalyst and substrate’ [75, p. 126]. Along these lines, we have inferred a receptor affinity distribution (RAD) broadly applicable to the immune and olfactory systems via a close analogue of string matching [65,79]. This portrayed a Poisson distribution, which in GARD applications [54] was approximated by a lognormal distribution [80]. For enhanced rigour, the inferred distribution was verified by meta-analysis of published data from diverse experimental systems, including phage display libraries, hapten–immunoglobulin interactions and enzyme–substrate recognition [81]. Not less important, when published mutually catalytic values of lipids from Fendler [82] were analysed, a similar distribution was observed [55]. This provided support for applying a functional form derived from equilibrium values (affinities) to catalytic (rate enhancement) values as done in GARD [53,54] (figure 3*a*,*b*).

In parallel, a completely different way to assign GARD catalytic values has also been explored. This was performed in the framework of a real-GARD (R-GARD) embodiment, which allows one to follow the growth and reproduction of assemblies composed of true phospholipids and cholesterol, using experimentally measured kinetic values [60]. The mutually catalysis terms were derived via mass action law, taking into account realistic molecular parameters for lipids (integrated from 16 sources [60]), including surface area, charge, ability to form complexes with neighbouring molecules and intrinsic curvature. This closer-to-nature model fully confirmed the standard GARD dynamics, including homeostatic growth and composome emergence. Thus, key GARD conclusions show concordance between a model that uses distribution-derived rate-enhancement parameters and that which employs parameters based on the physico-chemical behaviour of true molecules. R-GARD also provided new insight, e.g. that variations in the hydrophobic chain length influence the effective vesicle reproduction rate. This may relate to a finding that small concentrations of long-chain lipids assist the formation of vesicles primarily composed of short-chain fatty acid [83].

## 5. Replicating composomes

The foregoing sections dealt with broadly defined attributes of mutual catalysis that underlie homeostatic growth and compositional inheritance. It is now necessary to further probe the mutually catalytic dynamics of GARD. Employing the lognormal distribution with appropriate parameters [54], we can follow the simulated time-dependent dynamics of compositional transitions—a trajectory in the compositional *N*_G_-dimensional space (figure 2*b*). In these simulations we assume a well-stirred setting, whereby each molecule may encounter all others within a fast collision scenario, so one ‘can neglect any spatial correlations … and concentrate solely on the molecules' abundances’ [84, p. 400].

We discovered that in a typical simulation, considerable segments of the trajectory show little or no compositional inheritance, which we denote ‘drift’. Only when the simulation path happens to reach certain specific neighbourhoods in compositional space, does homeostatic growth and compositional inheritance emerge (figure 2*b*). These neighbourhoods in compositional space, constituting specific dynamic states of a molecular assembly are defined as composomes [47,54,63]. For different sets of catalytic parameters *β_ij_*, drawn from the same statistical distribution, different simulations portray between one and seven different composomes [61]. During the simulation, one can observe transitions from one composome to another, mediated by series of mutation-like compositional changes (see §6.2). The simulation path may encounter the same composome time and again, often in a somewhat different configuration. The similarity among different composomes is assessed via a dot product of the relevant compositional vectors, representing the angle between them. This procedure makes it possible to define being in a composome state at a given instance, as well as to define clusters of similar composome instances along a simulation, which are termed ‘compotypes’^2^ [64,85–87].

The functional form and parameters governing the distribution underlying the GARD mutual catalysis matrix play a key role in deciding whether or not compositional inheritance is seen. Thus, if the distribution used is normal rather than the experimentally faithful lognormal distribution, compositional inheritance is hardly observed [56]. Further, even just changing the parameters of the lognormal distribution may lead to drastically different behaviours, ranging from high heritability, to a state in which most assemblies undergo random split without information transfer [56].

Appropriate kinetic parameters are not the only necessary condition for the emergence of replicating composomes. We have shown that assembly size has a decisive influence on composome emergence. Even for optimal rate-enhancement parameters, if the assembly size (*N*_MAX_—immediately prior to fission) is sufficiently greater than the repertoire size (*N*_G_), no composomes appear. The constraint that needs to be obeyed is *N*_MAX_ ≤ *N*_G_ reflecting a ‘Morowitz boundary’ [88], based on Morowitz's showing that the transmissibility of information through direct inheritance of a molecular composition is related to the size of the assembly and the diversity of its molecular species [89]. Such constraints have important implications regarding the type of amphiphile assemblies that might show effective GARD reproduction capacity. In an example of a repertoire of *N*_G_ = 100, composomes would appear only in micelles, whose sizes are compatible with *N*_MAX_ = 100 [90]. With a much larger amphiphile diversity, say *N*_G_ = 10^6^, likely to prevail at life's origin [45], much larger assemblies, such as small (0.2 µm) vesicles (a size consistent with total molecular count of 10^6^), might portray replication/reproduction behaviour. We note that GARD dynamics of such large molecular counts has not been explored to date, yet such large counts are relevant to GARD's evolvability and emergence in a planetary context (see §§ 7.2 and 13).

Despite superficial dissimilarities, GARD shows a striking resemblance to Dyson's acclaimed origin of life model [8, p. 50]. Dyson defines ‘an abstract multidimensional space of molecular populations. Each point of the space corresponds to a particular list of molecules'. These lists map precisely to GARD's compositions, and the multidimensional points to GARD's compositional vectors (assembly compositions). Dyson then specifies that ‘The population is confined in a droplet, as Oparin imagined it’—a simile of a GARD lipid assembly. He further describes the molecular events that may take place: ‘The population of molecules within the droplet can change from moment to moment, either by chemical reactions within the populations, or by reactions incorporating small molecules from the medium or by reactions rejecting small molecules into the medium’. This is very similar to GARD dynamics, including the entry and exit of lipid monomers in basic GARD, and covalent transformations in metabolic GARD (M-GARD, §11.1). Finally, Dyson provides equations that describe the generalized time-dependent behaviour of the molecular populations, and asserts that ‘The population thus evolves in a stepwise and stochastic fashion over the space of possible states', and proposes to focus on populations ‘that persist during evolution over long periods’, calling them quasi-stationary states. These states strongly resemble GARD composomes and their homeostatic growth. Further comparison of the three models for mutually catalytic networks Dyson's, Kauffman's and GARD has appeared [53].

### 5.1. Catalytic closure and homeostatic growth

Extensive analyses have been recently devoted to a more formal definition of the original Kauffman model for autocatalytic sets, by Hordijk *et al.* [11,39]. These authors consider a network of catalysed chemical reactions, calling it reflexively autocatalytic if every one of its reactions is catalysed by at least one of the included molecules, and food-generated (F-generated) if every reactant can be constructed from a food compound set via included reactions. Reflexively autocatalytic and F-generated (RAF) then denotes systems that fulfil both conditions. ‘Thus, an RAF set formally captures the notion of “catalytic closure”, i.e., a self-sustaining set supported by a steady supply of (simple) molecules from some food set’ [11, p. 3087]. The authors further strive to define the exact conditions under which an RAF gets generated, including via the influence of increasing the complexity of the constituent molecules, and seek the parameter values that will ensure catalytic closure.

It is interesting to ask whether GARD composomes constitute an RAF system. To perform such an analysis it is necessary to take into account a key difference in definitions between Kauffman/Hordijk autocatalytic sets and GARD sets. The former rest on a binary classification of reactions as ‘catalysed’ or ‘not-catalysed’, while the latter (as suggested by its name) uses a graded scale for the magnitude of the catalytic effect. GARD parameters are embodied in the *β* matrix, with all elements being non-zero positive values. This state of affairs, including the lognormal distribution of *β* elements is aimed to capture the realism of biochemical mutual interactions, in contrast to the more symbolic binary definition used in the Kauffman model.

Taken at face value, as all entry–exit reactions are catalysed, and as every one of the *N*_G_ molecule types in the simulation also belongs to the food set, it follows that every GARD lipid assembly is an RAF. To allow a more discerning interpretation of this seemingly trivial verdict, it is possible to use a judicious catalytic intensity cut-off, below which the *β* elements are set to zero (no catalysis), as described [85,91] and indicated graphically in figure 3*c*. With such a modification, most randomly formed GARD compositional assemblies will not be RAF. On the other hand, as the compounds belonging to a composome show overlap with communities within the *β*-matrix-defined subsets of more tightly linked network nodes [27,92] (see §8), it follows that composomes are much more likely to be RAFs. If true, GARD kinetic simulations could be used, in parallel to the published analytic algorithm [73], to distinguish between RAFs and non-RAFs.

As said, without the above-mentioned cut-off, every GARD assembly is an RAF, and all such assemblies are catalytically closed. But non-composomal assemblies (drift) may be described (stretching the original definitions) as ‘weak RAFs’ with ‘weak catalytic closure’. GARD dynamics, en route from randomly seeded assemblies to composomes may be regarded as moving from ‘towards RAF’ to RAF, with gradually enhanced catalytic closure. The same progression appears in Kauffman's autocatalytic sets, which approach the RAF status along the synthesis of increasingly complex peptides, until the system undergoes the abrupt phase transition to catalytic closure [93].

A GARD composome by definition shows homeostatic growth, the hallmark of a replicating mutually catalytic network. Does an RAF system always show homeostatic growth, and therefore, reproduction? The answer is not straightforward, because RAF is not an explicit kinetic model. It focuses on the statistical parameters governing the network connectivity, including the gradual individual molecule complexification leading to catalytic closure. It does not address the concentrations of the different molecular species and their time-dependent changes. Therefore, in its present form the RAF model appears not to include the quantitative variables necessary for assessing growth with concentration invariance.

It is important to delineate some further differences in the properties of the two models. In contrast to GARD, RAF lacks an explicit mechanism for obliging the molecules to remain close to each other, such as inter-amphiphile attraction, as further discussed in §10. Another difference is that GARD assemblies, unlike RAF, feed upon self-accretion of environmental molecules without constraints on how complex they might be, maintaining catalytic closure all along. Finally, RAF begins with very simple molecules, e.g. single amino acids, and undergoes a gradual increase in molecular complexity (e.g. via oligopeptide elongation), so as to attain catalytic closure. By contrast, basic GARD lacks an endogenous molecular complexification path, similar to peptide elongation, to make further evolutionary progress. This has begun to be changed via the incorporation of covalent chemistry in M-GARD, as described in §11.1.

### 5.2. Compositional information

**Figure 4.**
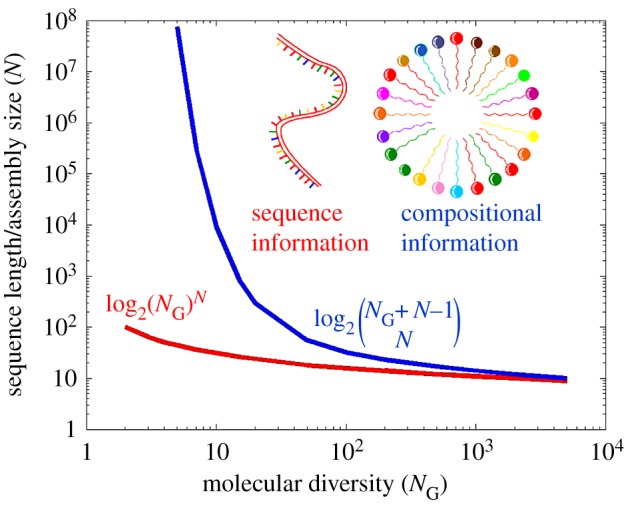
The sequence length or assembly size (*N*) that are required for encoding 100 binary bits by a polymer with sequential information (red line) or an assembly with compositional information (blue line) as function of the size of the molecular repertoire (*N*_G_). Values are based on the combinatorial formula shown for compositional information [94] and on the standard Shannon formalism [95] for sequential information, both in a case in which the frequencies of all monomers are equal. Evidently, at low *N*_G_, sequence information is a much more efficient encoder, but at high *N*_G_ (relevant to early life) the two information types become asymptotically equal. Adapted from [47].

A cornerstone of the GARD model is its reliance on compositional information. Just like sequence information, compositional information may be quantitated (figure 4) [94,96]. In analogy to the fact that all sequences of a given length *N* and an ‘alphabet’ size *N*_G_ contain the same amount of sequence information irrespective of actual sequence, all compositional assemblies of total molecular count *N* and an alphabet of *N*_G_ have equal compositional information. But just like the fact that in evolved organisms the sequence of RNA spells out translated functions, a specific composition can make the difference between drift and an effectively replicating composome. In this respect, we regard the composition of a GARD assembly as equivalent to a genome, hence ‘compositional genome’, which is the source of the term composome. The dynamic functionalities that depend on the composition may be regarded as a rudimentary phenotype (see §7.1). Such compositionally affected traits have been recorded in other chemical systems, supporting the realism of our model. Thus, a vesicle's lipid-composition has been shown to affect dye encapsulation efficiency [97] or vesicle's structure [98], and genetic/evolutionary algorithms have been applied to evolve vesicles' compositional formulation [99,100].

In the realm of GARD, we often use the terms ‘compositional assemblies’ and ‘compositional information’. These are equivalent to the RNA world terms ‘sequential biopolymers' and ‘sequence information’. However, strictly speaking, compositional and sequential information are not mutually exclusive. Thus, a compositional assembly may contain sequential molecules, such as peptides. By the same token, the set of all mRNAs in a cell is often dealt with compositionally, as in the realm of transcriptome analyses [101]. So in a final account all biomolecules embody both sequential and compositional information, with different functional readouts for each information type.

In the same vein, the utility of compositional information is highlighted in a paper [96, p. 4048] on a modified quasi-species sequence-based model, which focuses on the evolution of monomer frequencies within a polymer. This model assumes ‘that molecules composed of the same number of monomers of each type are equivalent, i.e., possess the same replication rate, regardless of the particular positions of the monomers inside the molecules'. Employing the same definitions for compositional vectors and compositional information as in GARD (§3), the enhanced simplicity of the model allows a more thorough analysis of the replication landscape.

In contrast to RNA, mutually catalytic assemblies belong to Monomer World [102]. While, as said, such monomers may be small sequential entities themselves, their replication is not necessarily based on strict templating of sequences, but on more generally disposed mutually catalytic interactions. Thus, while the exact molecular structure of each compound governs the interactions within the catalytic network, the individual molecules do not necessarily self-replicate by the rules of template complementation.

A GARD lipid assembly with the appropriate mix of molecules is a composome, having catalysis-governed growth–fission dynamics that results in the generation of faithful progeny (figure 2). Formally, this exact definition applies also to an assembly with only one type of amphiphile (*N*_G_ = 1). Indeed, such a homogeneous assembly will grow and split, and will generate absolutely exact compositional progeny. Such an assembly can form either in a homogeneous environment, or when a single compound is a very strong autocatalyst for molecular joining (cf. [103]). However, in either case, the group of homogeneous assemblies will have no diversity, hence it will not support dynamic variability, selection and evolution [61].

## 6. GARD protocells

A GARD composome maintains its compositional information largely unchanged for many growth–split generations, thus effectively portraying reproduction. But Morowitz and Deamer, in a pioneering paper about protocells [104, p. 281], write: ‘Here we discuss an alternative system (to RNA replicators) consisting of replicating membrane vesicles, which we define as minimum protocells’. This leads to the bold but inevitable conclusion that in certain cases, namely in the composomal state, GARD heterogeneous vesicles *are* protocells, significantly less minimal than the homogeneous vesicles alluded to in [104]. This notion is augmented by Dyson's comment [8, p. 38]: ‘As soon as the garbage-bag world begins with crudely reproducing protocells, natural selection will operate to improve the quality of the catalysts and the accuracy of the reproduction’. The clear message is that GARD's advent of a crude replication capacity marks one possible first step in the long evolutionary journey of a minimal protocell towards the last universal common ancestor (LUCA, see §§7.2 and 13).

A scenario that has been studied by Szostak for nearly two decades is the ribozyme protocell [66, p. 388, 105], described as follows: ‘Our simple protocell will consist of an RNA (ribozyme) replicase replicating inside a replicating membrane vesicle’. In addition, the protocell includes ‘a ribozyme that synthesizes amphipathic lipids and so enables the membrane to grow’. We note that the concomitant spontaneous emergence of both specialized ribozymes, one of which self-replicating, is unaccounted for, and is admitted to be a primary challenge of the model. This is over and above the often described hurdles for the abiotic appearance of RNA monomers and polymers [5,12]. A final weakness of the ribozyme protocell is that there is no demonstration that all the components will replicate at a proportional rate, leading to homeostasis.

Why is this protocell (and other instances thereof) assumed to contain RNA? Perhaps because of the unabated conviction, based on present-day life characteristics, that nothing but RNA can replicate information, and that pure lipids cannot transcend their traditional role in compartment formation. The advent of an unorthodox form of information, embodied in lipid assembly composition, along with physico-chemical demonstration that such information can be maintained and propagated, should eventually lead to a paradigm shift. This conviction is echoed in Dyson's words, which address his modelled crudely reproducing protocells: ‘It would not be surprising if a million years of selection would (then) produce protocells with many of the chemical refinements that we see in modern cells' [8, p. 38].

### 6.1. Fitness landscapes and attractors

**Figure 5.**
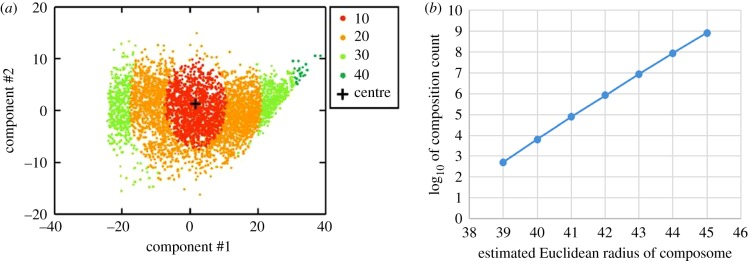
Estimating the count of different compositions in a composome. (*a*) Principle component analysis of a sample of approximately 10^4^ compositional vectors, each representing the composition of a GARD assembly from a constant population simulation at steady state. All the assemblies belong to the single compotype (a group of similar composomes) that emerges in this simulation. Colour is according to Euclidean distance from the compotype's centre of mass (black cross), with the shown scale denoting the maximum of each range. GARD parameters *N*_G_ = 100 and *N*_MAX_ = 100 were used. Figure reproduced from [62]. (*b*) An inferred trend line that provides a crude estimate of the composition count for different maximal Euclidean radii. As seen in (*a*), the compotype extends to a Euclidean radius of about 40. But as sampling may have been incomplete, and the trend represents a single compotype, an explored range up to radius of 45 is shown. This yields a crude estimate of 10^6±3^ for the total count of different compositions within a composome. The total number of possible compositions for the given values of *N* and *N*_G_ is computed by the formula in figure 4 to be 4.5 × 10^58^.

As described, in GARD a very small minority of all compositions belong to composomes. This translates to a very sparse fitness landscape, with very few ‘islands’ of effective reproduction in an ocean of ‘sterile’, non-reproducing compositions (drift). In a very crude assessment, only 10^3^–10^9^ out of 10^18^ possible assemblies actually belong to a composome (figure 5).

Despite such sparse fitness landscape, typical GARD simulations show that the internal kinetics leads from a completely random composition to a composome in a relatively small number of growth–split cycles. This is inferred from the initial slopes of compotype emergence in reactor simulations [64, appendix A], electronic supplementary material), exemplified in figure 7. Further, transitions from one composome to another in multi-composome *β* matrices are also quite fast (figure 3*d*).

This behaviour suggests that GARD composomes are attractors in compositional dynamics, as discussed [62,106], conforming to the definition of ‘a set of numerical values toward which a system tends to evolve, for a wide variety of starting conditions' [107, p. 113]. The intuitive kinetic rationalization behind this attractor behaviour is that for a randomly generated compositional assembly, molecules with weak total incoming catalysis (summed over an entire *β* matrix column, i.e. over index *i* in *β_ij_*) will be weeded out upon growth and split, while those receiving stronger overall catalysis will be gradually boosted. Small fluctuations towards the right composition will be catalytically augmented (see §9) so as to allow the catalytic network to reach composomes with surprising effectiveness. Composomes as attractors are further discussed in [106].

We note the intimate relationship between attractor behaviour and the linear algebraic analysis of the *β* matrix. As previously described, the solution of a linearized GARD equation points to the *β* matrix's eigenvector with highest eigenvalue, representing a canonical composome [27]. This serves as an attractor reached upon incessant assembly growth without fission and can be numerically computed [27,56]. However, this composome is never reached in the more bio-realistic simulations that involve periodic splitting and lead to one or more non-canonical composomes. The recent advent of a method for pre-identifying such composomes given the *β* matrix [92] will be of great help in future GARD analyses.

### 6.2. Compositional mutations and selection

The term ‘compositional mutation’ signifies a change in the count of a given molecule type in an assembly [62]. Such mutations arise from statistical fluctuations in the catalysed reactions that govern assembly growth, or in assembly fission [59]. The mutations occur readily because of the facile random access embodied in non-covalent entry and exit of monomers. While compositional mutations in a lipid assembly appear analogous to sequence mutations in a biopolymer, the latter are much more energetically demanding. In a non-templating scenario, mid-chain sequence variations involve the breaking of two covalent bonds and the making of two others. The covalent energy barriers are advantageous, resulting in long-term stability of sequence mutations. By contrast, compositional mutations, with their low energy barrier, are considerably less stable. On the other hand, compositional mutations are much more suited for early life, where covalent catalysis is expected to be weak or absent.

As a result, a single compositional mutation in an assembly is rather short-lived and may easily revert. But in the compositional world the stability of information rests in a very different mechanism—the attractor dynamics of composomes (previous section). What gets preserved is the affiliation with a specific composome, not the individual change. Every one of the variant entities that belong to the composome is a legitimate carrier of the information to the next generation. Only when too many mutations sequentially occur, the GARD assembly exits one basin of composomal attraction, transiting to drift or entering another composome. This process is demonstrated in the takeover phenomenon in our constant population reactor (chemostat) simulations [64] (see §7.2). Such a transition is also analogous to sympatric speciation in simple living organisms, e.g. bacteria [108].

In undergoing selection as a cloud of similar compositions, composomes are in fact analogous to quasi-species, as we have shown [62], whereby ‘the target of selection is not an individual mutant sequence but the whole quasi-species' [109, p. 121]. Prominent examples for that are viral quasi-species [110]. But in many published simulations, a quasi-species ‘has one master sequence with superior fitness … and all other sequences have inferior fitness’ [111, p. 2]. Further, all mutants reproduce, but are different in reproductivity [112]. Both of these characteristics are different from those of GARD. Some quasi-species simulations do describe population genetics scenarios [111], such as mutation-induced transition from one master sequence to another. Notably, in clear contrast to what happens in many simulated quasi-species scenarios, in GARD the fitness landscape is chemically governed, and not assigned by hand.

A highly relevant topic in this vein is the phenomenon of error catastrophe, a hallmark of the quasi-species formalism. This addresses the deleterious effect of an excessively high mutation rate. Thus, it was stated [113, p. 164] that: ‘…when this limit (error threshold) is crossed, the population disorganizes and (is) unable to maintain the genetic information’. We have mapped the composome formalism to that of quasi-species, showing that the quasi-species-equivalent is always around a composome and not around random ‘drift’ assemblies [62]. We further demonstrated that a GARD composome may undergo an error catastrophe. An increase in mutation rate was simulated by a decrease of the free energy driving force for amphiphile joining (lowering *k*_1_ with unchanged *k*_−1_, thus decreasing the equilibrium constant *K*_eq_ = *k*_1_/*k*_−1_). This increases the propensity of highly mutated compositions, providing a thermodynamic view of the GARD error catastrophe [62]. Importantly, in the GARD realm, a series of compositional mutations would mark the temporary demise of a composome C1 but in the longer run would spell the subsequent dynamic emergence of another composome C2 [54].

## 7. GARD evolution

### 7.1. GARD phenotype and genotype

The core mechanism of selection is that a mutation in a replicating information carrier (genotype) somehow affects its capacity to generate its own copies. In present-day life, such a link is most often mediated by an encoded phenotype and selection acts via the phenotype. As many instances of the standard quasi-species model do not explicitly include a phenotype, methods have been proposed to invoke phenotypes implicitly. For example, in a modified quasi-species model [84, p. 400], selection is implemented based on the argument that ‘self-replication … consumes energy and substrates from the environment. These external resources are … not modeled explicitly … (but) the degree to which a macromolecule finds the resources necessary to self-replicate … is expressed in the replication coefficients’. It appears that the authors assume that each mutated sequence has its own sensitivity to resource availability, probably mediated by an encoded phenotype that varies in some correlation with the sequence mutations. Another case in point is the use of sequence–structure (folding) maps of RNA as a proxy for genotype–phenotype maps. In this case, the RNA molecule undergoes mutations that directly affect the folding phenotype which is subject to selection [114].

In life as we know it, such correlations are readily explained, e.g. via a mutated encoded protein. Nevertheless, importantly, for a nucleic-acid mutation to influence only the replication rate of that specific mutant, each mutant has to be enclosed in a different reproducing compartment (e.g. a virus particle), so that the phenotype-dependent fecundity of that compartment affects only the copying of the specific informational polymer contained within it. It is considerably more difficult to envision how, in a collection of ‘free floating’ sequential polymers, such a correlation will arise.

A unique property of compositional assemblies under the GARD scenario is that the composition (genotype) directly determines the assembly's replicative dynamic properties (phenotype). In a broader realm, this is exemplified by a correlation seen between a composome's restricted repertoire size *N*_MOL_ (the subset of environmental molecular types taking part in this composome) and the ecology-like population-growth rate in simulations of composomal populations [64]. This becomes possible because in GARD, the compositional genome of an assembly has direct kinetic influence on the efficacy and exactitude of homeostatic growth, hence on assembly reproduction. This allows the all-important correlation between mutations and reproduction to occur without a need for an intermediary, such as an encoded protein or folded functional RNA. Stated differently, GARD composomes represent both a genotype and a selectable phenotype, anteceding present-day biology in which the two are mostly separated. Arguably, this simplicity makes GARD lipid assemblies prime candidates for very early evolution.

### 7.2. GARD can evolve

The foregoing sections portray evidence for the capacity of mutually catalytic networks embodied in lipid GARD assemblies to undergo self-reproduction, bequeathing their compositional information. This conviction is shared both by proponents [15,70,115] and critics [27,116]. But self-sustainment and replication/reproduction are only one of the two essential characteristics of life by the NASA definition, the other being a capacity to evolve. The question asked is whether mutually catalytic networks, and their specific GARD embodiments, pass this test.

Support for the evolvability of composomes can be inferred from a paper on evolution before genes [74, p. 2]. The authors claim that while mutually catalytic networks in their entirety cannot evolve, subnetworks thereof, called cores or compartments, can. These subnetworks are defined as ‘more strongly connected autocatalytic cores', proposed to be ‘units of heritable adaptations in reaction networks … (That) can be viewed as a chemical network genotype’. It turns out that the definition of cores/compartments fully overlaps with that of composomes (see §8), as has actually been explicitly stated in another paper by the same authors [27]. These cores/compartments are identical to what in graph theory is called communities, whose relevance to GARD is discussed in §8 and in [92].

A biological evolutionary process entails selection for a variant information carrier in response to environmental challenge. An example is a bacterium taking adaptive advantage of a carried DNA allele that allows it to feed on a new environmental compound. The purported chasm between the standard quasi-species model and population genetics may underlie the fact that only a few RNA quasi-species analyses address such a scenario [111,114,117].

GARD has a unique fitness landscape, characterized by relatively few sharp peaks corresponding to the replicating composomes. As mentioned, while in quasi-species analyses the fitness peaks (often just one) are decided arbitrarily, in GARD such peaks are dictated by endogenous reproduction dynamics, the outcome of a network of chemical interactions. It, therefore, seems advisable to probe GARD's capacity to evolve along its own terms, rather than via a quasi-species formalism as attempted [27] and employ approaches directly related to biological evolutionary logic.

A rewarding aspect of GARD is that its compositional genome interacts directly with the chemical environment, so that a translation device is rendered unnecessary. So GARD evolvability should best be tested by simulations that take advantage of this merit, i.e. by changing the simulated chemical environment. This is as opposed to changing the *β* matrix to provide a small advantage to specific compositions as done [27] which not unexpectedly leads to negligible effects because of the attractor nature of composomes (see §6.2).

**Figure 6.**
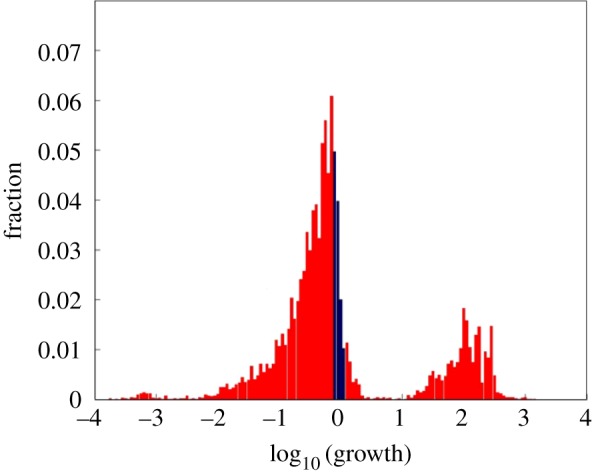
The consequences of completely depleting a single compound from the operative repertoire of *N*_G_ = 30 compounds [85]. We simulated GARD traces (as defined in figure 2 and [118]) with 500 generations for 1000 different *β* matrices. For each trace (as defined in figure 2*b*), we performed environmental compound depletions for each of the *N*_G_ compounds, by setting to zero an entire row and column of the *β* matrix, representing the given molecule. Subsequently, the assembly growth rate before and after depletion was compared. For multiple composome traces, the pair of the maximal compositional similarity between the composome before depletion and that after depletion was examined. The horizontal axis is normalized growth rate, the ratio of growth rates after and before depletion. *X*-axis values between 0.79 and 1.25 are marked in blue and their very high propensities are attenuated to allow a better view of the minority cases outside this range. Among all depletion instances approximately 10% diminished their growth appreciably, and approximately 1% showed enhanced growth rate in the range of X3–X300. This is interpreted as indicating that the composomes involved had higher fitness in the modified environment. We note that in the original paper [85], the induced changes were referred to as network mutations, but these are formally fully equivalent to compound depletion from the environment. The figure is adapted from [118].

We performed a simulation study with many repetitions with different *β* matrices, asking what are the consequences of completely depleting a single compound from the environment repertoire of *N*_G_ = 30 compounds [85] (figure 6). A majority of depletions had only a small effect on the composome growth rate, but approximately 10% of them diminished growth appreciably, and approximately 1% rewardingly showed up to ×300 enhancement of the composome replicative growth rate. This indicates that the composomes involved had higher fitness in specific modified environments. That only a minority of the environmental changes had an appreciable effect is much in line with standard evolutionary dynamics.

**Figure 7.**
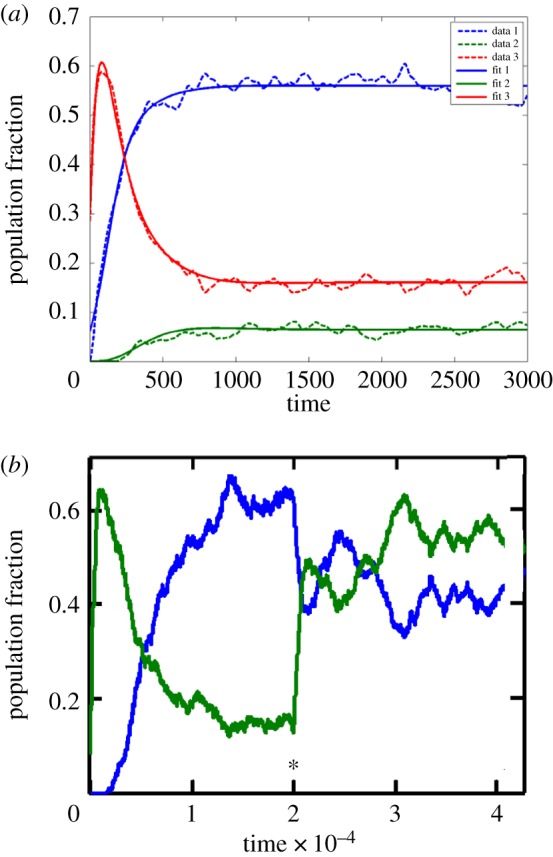
(*a*) Time dependence of the propensities of compotypes (composome clusters) within a constant population reactor, for three compotypes (C1—red, C2—blue, C3—green). Takeover is observed between C1 and C2, the latter overcoming the former, becoming dominant at steady state. Simulation data are depicted by the broken line and a numerical fit to an ecological logistic equation (cf. [61]) is depicted by the solid line. Time is given in units of split events. Modified from [64, appendix]. (*b*) Dynamics of composome propensities in a reactor simulation as in (*a*), for a *β* matrix with two compotypes, C1 (blue) and C2 (green). In the standard GARD kinetic formalism (equation in figure 1), *ρ_i_* represents the environmental concentrations for the *i*th compound. In most simulations performed by us, including that shown in *a*, *ρ_i_* is set to be equal for all compounds. Here we modified this condition, whereby at time point 2 (20 000 splits, marked by asterisk) the external concentrations were changed to *ρ*_*i*_(*t*=2)=0.9*ρ_i_*(*t*=0)+0.1*n_i_*(C2) where *n_i_*(C2) is the *i*th element in the composition vector of compotype C2. This has the effect of a small degree of biasing of the molar composition of the environmental chemistry towards the composition of C2. The result is that C2 rose from being a minority at steady state to slightly above C1 after the environment shift at *t* = 2 (lasting to the end of the simulation). Time is given in ten-thousands of split events.

In another evolution-related study [64], we followed the fate of a population of 1000 GARD assemblies in a constant population reactor. In such simulations, one observes several compotypes (clusters of similar composomes) of a given *β* matrix at the same time, as well as drift (weakly reproducing or non-reproducing assemblies). This set-up reveals the time-dependent relative abundances of different compotypes, reflecting properties such as growth rate, reproduction fidelity and compotype lifetime [61]. One of the interesting phenomena revealed is ‘takeover’, whereby compotype C1 may be dominant transiently, but at long-term steady state, another compotype C2 becomes more prevalent (figure 7*a*). While this may seem preordained via the elements of the *β* matrix, hence not a true evolutionary phenomenon, it provides insights into modes of compotype competition. This is instrumental in simulations of more complex and life-like GARD analyses, as described below.

We used the same reactor analysis tool to examine the effect of changes in the external chemistry that are broader than single compound depletion (Fouxon *et al*. 2014, unpublished data). In a preliminary set of simulations (figure 7*b*), we modified the external concentrations, biasing them towards the concentration vector of an initially unfavourable compotype. This resulted in a takeover by the targeted compotype, generating a new reactor steady state. This result complements the above-mentioned takeover reactor experiment showing GARD's capacity for long-term changes, including those occurring in response to environmental cues. Similar results were also reported in [58].

A third GARD reactor simulation was performed with an R-GARD embodiment [60] (described in §4), where we explored assembly competition for a finite supply of amphiphiles [119]. The simulation is initiated with the standard concentrations of external amphiphiles and ends with complete depletion of the environmental molecular supply. In such simulations, we were able to transiently observe complex kinetic behaviour, including inter-assembly competition. This demonstrated that a gradually depleting finite environment in GARD can portray evolution-like behaviour.

**Figure 8.**
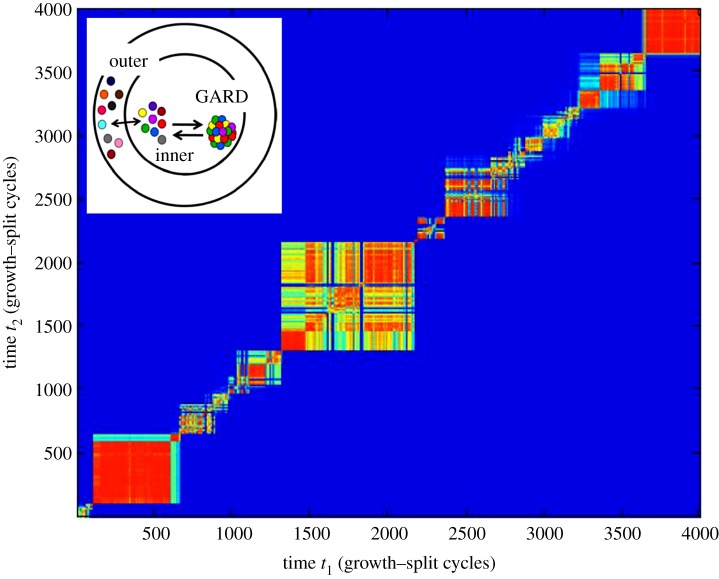
Composome emergence in the Universe-GARD (U-GARD) variant model of GARD [86]. Design is as in figure 3*d*. Assemblies undergo growth–fission cycles in the inner environment, obeying the GARD dynamics. Compound types exchange between the inner and larger outer environment (inset). The simulation shows ongoing emergence of new composomes along the time axis. This dynamics is consistent with open-ended evolution in the time interval simulated, as contrasted with the more standard GARD behaviour with a limited number of composomes that repeatedly appear. Adapted from thesis [120].

One possible evolutionary-significant GARD complexification is an increase of the molecular repertoire *N*_G_ without changing the maximal assembly size value (*N*_MAX_ = 100). This direction has been explored via a yet another GARD version termed universe-GARD (U-GARD) [86]. In this, the immediate environment of the assembly with *N*_G_ compounds is embedded in a larger outer ‘universe’ with a larger molecular repertoire (figure 8). The external universe may be realistically regarded as mimicking molecules that are either absorbed on mineral surface or contained in neighbouring mineral pores. Also, it may be interpreted as related to incomplete mixing in the aqueous environment. A diffusion process is assumed to allow entry of new molecule types from the external to the internal reservoir, subject to some constraints [86]. The results shows continuous emergence of new compotypes along the time axis (exemplified in figure 8). This dynamic is consistent with open-ended evolution in the time interval simulated, as contrasted with standard GARD behaviour with a limited number of composomes that recurrently appear. The dichotomy between repeatable dynamics and open-endedness is considered one of the hallmarks of a transition from chemical evolution to biological evolution [71].

A modified version of the above exploration is a consideration of the standard single environment, but with a substantial molecular repertoire, say *N*_G_ = 10^4^ (versus 10^2^ typically used), with unchanged assembly size *N*_MAX_ = 100. Such a state of affairs is in line with prebiotic chemical realism, with small assemblies embedded in a chemically diverse environment [88]. The following describes inference, not yet substantiated by simulations. If we use our standard reactor scene, the initial combinatorically generated 1000 assemblies will obligatorily be vastly different from each other, nearly orthogonal [54,121,122]. We posit that the initially appearing composomes will be suboptimal because of the 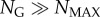 condition, which hinders assemblies from directly reaching the best possible composomes drawn out of the entire 10^4^ compound type repertoire. This is because a given assembly can only harbour less than 1% of all possible molecule types at any given time. We anticipate a long process of ‘annealing’ with consecutive compositional changes, whereby increasingly better composomes will become dominant by takeover events, approximating open-ended evolution for a rather long time interval. Some simulations that support such predictions appears in figure 11*c*, where some of the 29 composomes shown in a dimer-GARD situation appear fast, but other take much more time to first appear. These are transitions from one fitness peak to another, a property usually associated with true biological systems [71]. Because some of the future analyses may require prior knowledge of which composomes might appear, a recently published algorithm that can predict candidate composomes for any *β* matrix [92] becomes instrumental.

Finally, it is important to highlight the criticism that as long as GARD is limited to non-covalent chemistry, its capacity to show full-fledged evolutionary characteristics is limited [27]. For this reason, we have maintained a long-term effort to study GARD versions that include various types of covalent modifications, as described in §11.1. The inclusion of covalent synthesis in GARD versions is equivalent to combinatorically effected repertoire enhancement, hence likely to gradually portray better and better evolutionary openness. Progress along these lines will further improve GARD's stand regarding a full-grown capacity to evolve.

## 8. Repertoire diminution

**Figure 9.**
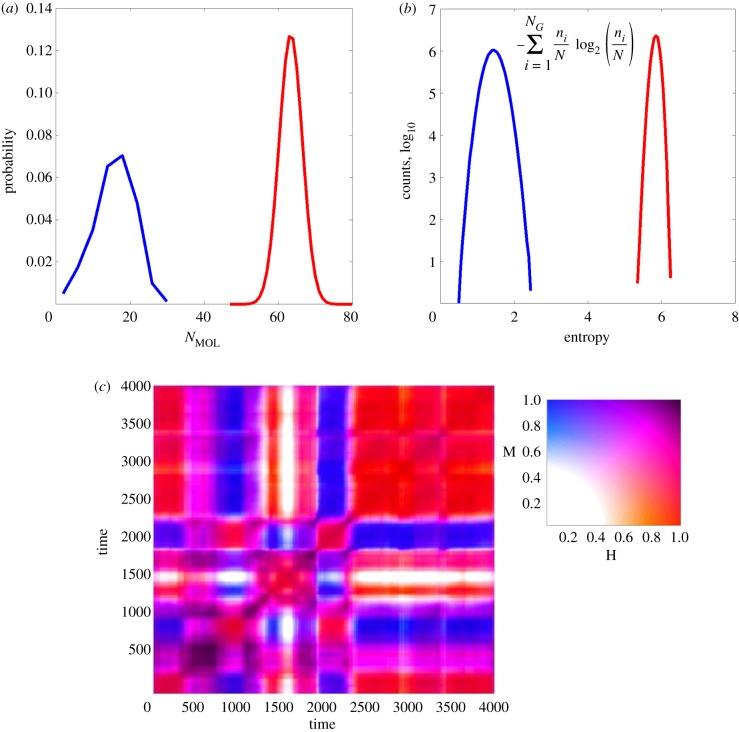
(*a*) The distribution of the molecular repertoire size *N*_MOL_ in composomes (blue) and in randomly formed assemblies (red). The composomes analysed are from [64] and with parameters as in figure 8. For random assemblies a calculation was performed, generating 10^7^ compositional vectors with repertoire *N*_G_ = 100 and *N*_MAX_ = 100. (*b*) The distributions of entropy, computed by the formula shown. For composomes (blue), the curve is based on data derived from [61], showing nearly a power law distribution of *n_i_* values for the data used in *a*. The entropy distribution for random assemblies (red) is based on the same data as in *a*. (*c*) Result of simulation of Chiral GARD as described [123]. The parameters used are *N*_MAX_ = 300 and *N*_G_ = 200, the latter including 100 different amphiphiles, each in two enantiomers d and l. This parameter range permits both information transfer and intrinsic enantiomeric selection. Inset: two-dimensional colour scheme displaying the chiral relation between two compositions. *H* is the dot product similarity to another composition, *M* is the dot product similarity to the exact antipode of the composition. The main figure has the same design as figure 3*d*, with time steps representing growth–split cycles. Red diagonal squares show composomes, with many consecutive time points having strong mutual similarity. Off-diagonal blue rectangles signify that the crossed composomes are enantio-selected but their compositions are antipodally disposed. Purple diagonal squares are racemic composomes. Figure adapted from [124].

GARD's internal kinetics leads from a random composition to composomes. This dynamic process involves a considerable diminution of the molecular repertoire in the assembly. Sampling statistics shows that a random GARD assembly of size *N*_MAX_ = 100, formed from an extraneous molecular repertoire *N*_G_ = 100, will have an actual internal repertoire of *N*_MOL_ = 63.4 ± 3.1 with molecules types showing a Poisson distribution of their counts. By contrast, when the assembly is in or near reproducing composomes, the average internal repertoire drops to *N*_MOL_ = 15.9 ± 5.5 [64], with the rest of the compounds absent (figure 9*a*).

Molecular repertoire diminution may be viewed as internal molecular selection, happening as a consequence of reaching a state of strong catalytic closure, whereby molecular species belonging to a particular mutually catalytic sub-network become dominant within the assembly. This is distinct from the evolution-related true selection of an entire composome versus others. That composomes have diminished repertoire as compared to the external chemical diversity is in line with the network theory describing ‘communities’, defined as ‘groups of vertices having higher probability of being connected to each other than to members of other groups’ within the network [125, p. 1]. The same phenomenon is also described as ‘hidden compartments’ of the GARD *β* matrix [27].

Rewardingly, repertoire reduction is also a hallmark of the evolutionary process that led to present-day living cells. Cells typically contain a very small fraction of all possible low molecular weight (monomeric) compounds. Thus, while molecules with up to 30 atoms of the elements carbon, nitrogen, oxygen and sulfur show a crudely estimated multiplicity of 10^23^ [126], only 2600 molecules are counted in a bacterial metabolome [127] and 114 000 in the human metabolome [128]. For smaller molecules with up to six non-hydrogen atoms (with the above elements and phosphorous), there are 2600 different molecules, but only 25% seen in any reported metabolome [129].

### 8.1. GARD enantioselection

One of the most gratifying consequences of mutual catalysis-based repertoire reduction is its potential to underlie enrichment of one or the other enantiomer of chirally asymmetric molecules. That autocatalysis can underlie the selective amplification of one optical isomer versus the other has been established [130]. Extending this phenomenon and exploring it in a well-tested mutually catalytic network context, we have shown that such a network is capable of enantioselection. We simulated a chiral GARD assembly formed in an environment with *N*_G_ chiral compounds, each with an equal representation of both enantiomers (racemic mixture). When repertoire restriction occurred upon the dynamic transition into composomes, substantial enantioselection was observed. This phenomenon is traced down to the fact that some optical isomers may be completely absent under the small number statistics of a particular seeded assembly, leading to mutual catalysis-mediated symmetry breaking in chiral GARD [123]. This is akin to the proposed mechanism for symmetry breaking by stochastic chiroselective co-oligomerization in oligonucleotide systems [122]. We note that this implies that enantioselection could be one of the consequences of early reproduction in a mutually catalytic network, rather than an often claimed prerequisite for life's emergence [131]. This is in line with a recent assertion that homochirality in nature is a stereochemical imperative [132].

### 8.2. Entropy reduction

That composomes show a molecular repertoire diminution, with skewed distribution of molecular counts as compared to randomly seeded assemblies, implies that composome emergence involves a negative change of entropy (figure 9*b*). This is related to the reverse of the entropy of mixing, defined as the increase of disorder in a multi-component system upon transition from an unmixed to a fully mixed state [133]. In this, composomes capture an essential property of living cells, whereby order is produced within cells as they grow and divide. This, of course, is more than compensated for by disorder appearing in the surrounding milieu, a process fed by using externally supplied free energy. This state of affairs conforms to the resolution [134] of Schrodinger's paradox regarding how living cells avoid decay into chaos despite the second law of thermodynamics [135]. Schrödinger prophetically predicted DNA, whose idiosyncratic subunit arrangements encode the information needed to instruct orderly structure and function within cells [136], but it appears that mutually catalytic networks may accomplish the same feat.

What is remarkable about GARD compositional assemblies is that they represent a very simple model for how order emerges out of disorder. GARD composomes are not merely biased molecular mixtures: despite the fact that in the present model's embodiment they do not assume spatial organization, they spontaneously emerge as functional units—mutually catalytic networks. Even more impressive, these networks are shown to underlie a fission-supported reproduction-like process, allowing the assemblies to propagate themselves, converting additional material to a low entropy ordered configuration. In this, they seem to foretell higher forms of dynamic order seen in present-day cells, including self-organization, metabolism and biopolymer replication/encoding.

## 9. GARD is a dissipative system

A dissipative system is a thermodynamically open system, which operates away from equilibrium, embedded in an environment with which it exchanges energy and/or matter. Such a system portrays a kinetically controlled dynamic behaviour that corresponds to a steady state. The term ‘dissipative system’ was coined by Prigogine, who also defined ‘dissipative structures' as manifesting local self-organization away from equilibrium, hence with a capacity to maintain local low entropy [137]. In these systems, organization can emerge through a spontaneous breaking of symmetry, both spatial and temporal, that can lead to the generation of complex structures [138].

One possible mode of symmetry breaking is unmixing, whereby compounds of different types that are originally mixed with each other, get separated, leading to the lowering of entropy [133]. This is echoed by Prigogine as follows: ‘If we let two liquids mix … this is a typical example of situations described by an increase of entropy. On the contrary, in biological systems heterogeneity is the rule: inequalities between concentrations are maintained by chemical reactions and active transport’ [137, p. 110]. Such transitions from an equimolar mixture to a biased mixture, which happen in living dissipative systems, rewardingly also appear in GARD composomes. Prigogine provides a succinct description of how generally this becomes possible: the application of ‘specific non-linear kinetic laws beyond the domain of stability of the states showing the usual thermodynamic behaviour’. In this, he addresses his formal treatment of dissipative systems and structures [137]. In a similar vein, England [139] analyses the irreversible thermodynamics of a self-assembly process in which particles stick together to build up an assembly with internal structure and composition.

Do composomes, as simulated by GARD, constitute a dissipative system? The answer is definitely yes, as detailed in the list of criteria below.

**Figure 10.**
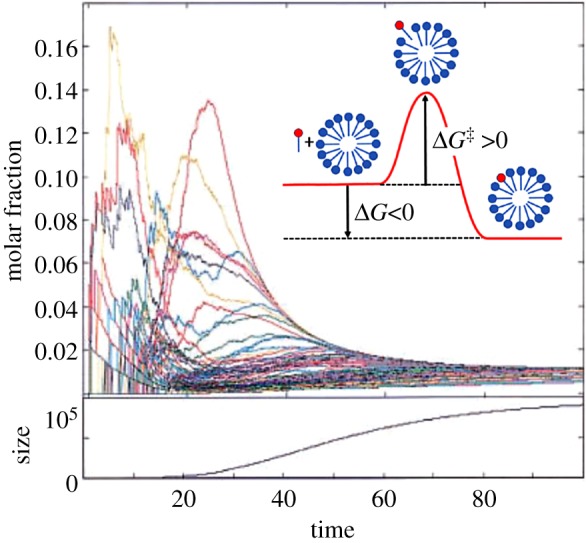
Evidence that GARD is inherently an out-of-equilibrium model. In the main figure, the time dependence of the molar fraction *n_i_*/*N* inside a GARD assembly is shown for each of the molecule types *A_i_* (different colours), as a function of time (units are intervals of 0.05 s of simulated time along a dynamic progression of the equation in figure 1). This simulation, unlike the more standard ones, takes place without fission and with a finite supply of molecules. In the early part of the time course, transient composome-like dynamics takes place, as evidenced by the differential molar fractions of certain molecule types. At later times, due to exhausted resources, all molecular species assume an identical molar fraction plateau, reaching equilibrium. Concentration equality at the plateau is expected, because in our simulations all external molecular concentrations are set as equal, and all thermodynamic constants (*K_i_* = *k_i_*/*k*_−*i*_, see equation in figure 1) are also set to be equal. By contrast, the transient is clearly out of equilibrium, as is the case for the exact same simulation conducted with fission and with unlimited compound supply [54]. Bottom: gradually increasing assembly size, reaching a plateau upon amphiphile depletion, where equilibrium is attained. Inset: Free energy diagram for assembly entry and exit of an individual amphiphile. The entry is associated with a negative equilibrium free energy change (Δ*G*, measuring approx. 20 kCal mol^−1^, [140]). The free energy of activation (Δ*G*^ǂ^) barrier may have to do with the obligatory unfavourable contact of the hydrophobic tail of a half-inserted amphiphile with the polar headgroups of molecules already within the assembly [140]. Figure is modified from [54].

(a) *Away from equilibrium*. This is evidenced by fact that if a GARD assembly is allowed to go to equilibrium, e.g. by restricting the external amphiphile supply, the assembly's internal concentrations go to values dictated by the equilibrium constants, and become decisively different from those in the composomal state [54] (figure 10). We note that amphiphile assemblies may also reach equilibrium when no external free energy input exists which leads to their occasional fission [28].
(b) *Kinetic control*. This is obvious from the GARD differential equations (figure 1), that involve forward and backward rate constants (for entry and exit of each amphiphile), as well as rate-enhancement constants (the *β* matrix) that control mutual catalysis. Such constants dictate the time-dependent transition from random concentrations to those of a kinetically controlled composome.
(c) S*teady state*. During time intervals at which a composome prevails, the assembly is at steady state regarding its amphiphile concentrations. These remain largely unchanged both during the fast molecular entry and exit upon growth, and also during the slower change that involves many growth–split cycles. We note that this single-assembly steady state is distinct from a GARD population steady state in a reactor (see §7).
(d) *Exchange of matter with environment.* A GARD amphiphile assembly continuously exchanges molecules with its aqueous environment. This happens whether the assembly is in a composome state or not. It is due to kinetically orchestrated exchange that a composome state is finally reached.
(e) *Exchange of energy with environment.* The non-covalent joining reaction is down the free energy gradient. In this specific context, the incoming amphiphiles serve as externally supplied high-energy molecules (figure 10). Their joining the assembly is associated with the release of heat to the environment. Fission ‘resets’ the assembly to its pre-growth size and its per-assembly energy state, and the act of fission, therefore, has to require energy investment from the outside.
(f) *Self-organization.* GARD composomes portray a special type of self-organization, different from the typical spatio-temporal patterns seen in experimental models in the realms of inorganic chemistry (Belousov–Zhabotinsky reactions [141]) or physics (Benard cells [142]). In analogy, GARD assemblies spontaneously undergo an unmixing transition, which is a compositional self-organization. And as mentioned, self-organization propagates to progeny as composomal reproduction takes place.
(g) *Local entropy decrease.* This clearly happens, as detailed in the previous section and figure 9*b*.
(h) *Fluctuations.* In dissipative systems, microscopic fluctuations are sometimes amplified, and finally, stabilize to a macroscopic structure [137,143]. Every set of chemical reactions can produce a qualitatively different behaviour [144]. This is faithfully represented in GARD, where fluctuations arise due to the small assembly size, affecting both the growth kinetics and the fission outcome. Such fluctuations are catalytically augmented, culminating in the appearance of a composome [54,88]. GARD enantioselection is a prime example (§8.1).
(i) *Attractors*. In one of his last papers [143, p. 486], Prigogine states that ‘What is of primary importance in dissipative systems is that they have attractors'. We have shown that composomes are attractors, and that they are distinctive in their reproduction capacity—attractors begetting others via its internal catalytic mechanisms.

In sum, GARD composomes are clearly dissipative systems, away from equilibrium, described by a well-defined, quantitative molecular-level kinetic model. GARD thus becomes part of an effort to follow Prigogine's guidance, seeking specific kinetic rules [137] that may have governed life's origin.

A profound question still awaits an answer: is being a dissipative system both necessary and sufficient for life's origin? ‘Necessary’ means that dissipative systems, as rigorously defined by Prigogine, must have been involved somewhere on the path between abiotic to biotic. There is a rather broad consensus about that, since life itself is clearly a dissipative system. The ‘sufficient’ condition is less consensual: if the answer is yes, we note that this does not mean that *every* dissipative system would lead to life. This review makes it very clear that that the kinetic details of a particular dissipative system must lead to a capacity to self-reproduce, as in the case of GARD. Such a capacity is one of many self-organizing behaviours that may be assumed by a dissipative system, as clarified in points (f) and (h) in the above list.

If the answer is no, it means that there is a need for ‘something else’, including potential modification of the foregoing definitions, for life to emerge. What might such changes or additions be? One case in point is a claim that steady state, as defined in the context of dissipative systems, is insufficient to cover the emergence of replication. A new term, dynamic kinetic stability (DKS) has been brought forth as ‘a stability kind that governs the evolutionary process for both chemical and biological replicators' [145, p. 185]. To prove the need for such a new kinetic concept, the author would have to quantitatively define this measure, and to demonstrate that the standard definition of steady state is insufficient. Both of these actions have not been adequately fulfilled (see two critiques following the main text in [146]). Others have voiced arguments countering the need to define DKS as distinct from steady state, whereby in complex/dissipative systems, the emergence of complexity actually covers replication [147]. Further, it was noted that the standard dissipative steady state formalisms apply not only to templating polymers, but even more generally, to biological development and evolution [134]. We further argue that GARD composomes exemplify the lack of need for a redunadant term like DKS, because GARD replicates (or reproduces), yet its entire dynamics is satisfactorily covered by the standard formalisms of out-of-equilibrium steady state kinetics. Finally, in case an argument is made that *sequence-based* replication is an exception, requiring a special term, we have pointed out [59, p. 236] that ‘a templating strand may be thought of merely as enhancing the incorporation of ‘correct’ nucleotides into a growing strand, in comparison to the basal (slower) incorporation of ‘wrong’ nucleotides. Thus, templating is a special case of catalysis, and DNA/RNA replication is, in some respects, part of a cell's (or protocell's) metabolic network’. All the above points substantiate the ineptness of a replication-specific kinetic concept, underscoring the idea that DKS is a redundant term that may generate unnecessary confusion.

Another vote for an insufficiency of dissipative systems for understanding life's origin is a statement [148, p. 211, 149] that dissipative structures ‘occur spontaneously according to natural “law” propensities and are purely physicodynamic’, but that they cannot fully account for life's origin because they ‘involve no steering toward algorithmic success' provided by ‘prescriptive information’. We assert that mainstream science pursues what can be understood via physical and chemical laws, and that any suggested exception belongs to the realm of pseudo-science.

## 10. Lipid world

As detailed in previous sections, GARD, as explored in most of our papers, is a lipid-based model. The widely acclaimed role of lipids in life's origin is summarized by Pohorille [150, p. 357]: ‘In most modern theories of the origin of life, it is postulated that protocells—self-assembled, membrane-bound structures that encapsulated the nascent metabolism and information molecules—emerged early in the transition from inanimate to animate matter’, as further reviewed [151]. In this vein, Chyba [3, p. 218] states: ‘The Darwinian view of life does not require compartmentalization … However … the unit of all contemporary life is the cell, and an obvious function of the cell membrane is to maintain macromolecular components within an enclosed microenvironment’. He then draws an analogy to prebiotic settings, echoed in numerous publications as reviewed [152,153]. However, constraining water-soluble molecules to an enclosed volume is not the only reason for considering lipids/amphiphiles as essential for the origin of life. Other valuable characteristics (Table 1) relate to the chemistry *within* the lipid bilayer. These features include spontaneous accretion of diverse and potentially interacting molecules from dilute solutions, and the facilitation of mutual interactions due to proximity effects and reduced dimensionality, as copiously indicated [29,67,115,154–156].

Further support for a broader-scope significance of the lipid world is found in papers by Zhang. In [44, p. 440], he emphasizes the advantage of facile environmental availability: ‘it is unlikely that under prebiotic conditions the complex and sophisticated biomacromolecules commonplace in modern biochemistry would have existed. Thus, research into the origin of life is intimately associated with the search for plausible systems that are much simpler than those we see today … these simple building blocks of life might have been amphiphilic molecules’. He also proposes [44, p. 445] a way out of the potential abiotic scarcity of more complex lipids: ‘…instead of lipid membranes, simple … lipid-like peptides of various lengths could form and self-organize into distinct vesicles and tubes that could act as naturally formed enclosure … stimulating their own synthesis and replication’.

In the framework of the GARD model, the importance of accretion constitutes more than just molecular joining and vesicle growth: the heart of this model's dynamics is mutual catalysis that governs the accretion rate of every individual molecule, a progression that leads to homeostatic growth. Not less important for GARD is the capacity of lipid assemblies to bud and fission, which allows progeny to form, and completes the cycle of compositional information transfer along generations. Fission is often described cursorily as relying on the intrinsic properties of the vesicle and on environmental shear forces [66], but a more detailed analysis has been provided by Solé [67, p. 282]. Describing growth and fission in single-lipid-type assemblies, the author perceptively comments: ‘Since no information is included in this system, no further evolution is expected to occur. But this might have provided the initial, functional subsystem … for subsequent protocells of higher evolutionary complexity’. This is the exact mission the GARD model strives to undertake, in the footsteps of Oparin's coacervates model [46].

The basic GARD dynamics occurs *within* the lipid phase. Even when embodied in vesicles, GARD's capacity to undergo catalysis-mediated reproduction does not depend on any lumenal content (except in M-GARD, see §11.1). So why is GARD implemented in a lipid assembly at all? The answer lies in the close molecular proximity imperative: mutual catalysis requires that molecules will easily find each other in a facile collision process. The lack of such was pointed out in a criticism [157, p. 349] of Kauffman's autocatalytic networks model: ‘There are huge kinetic transport issues … with 1 000 000 different types of molecules'—how to organize them ‘in such a way that the catalyst … will be in the right proximity to the necessary reactants’. This conundrum is evidently resolved in GARD, via its being amphiphile-based, whereby all network members find each other easily within the condensed lipid phase. Absorption to mineral surfaces or delimiting in a mineral pore [158,159], does not provide for the need to have the enclosure constituents as part of the replicating catalytic network.

It appears that the best, easily available, embodiment for a single-phase multiple-function scenario is lipid phases [155]. Amphiphiles spontaneously form aggregates of different size and shape, mostly fluid at a relevant range of temperatures, affording a facile arena for mutual interactions. Amphiphilic assemblies also provide the molecular heterogeneity needed for the diverse functions underlying mutual catalysis, as attested by a database that reports 30 000 different known lipid structures [160]. In this scope, much beyond ‘walls only’, my colleagues and I coined the term ‘Lipid World’ in the context of life's origin [55]. Support for many of the above notions is in the recent description of a similar lipid-based mutually catalytic scenario for the early evolution [161–163].

We further probe some properties of amphiphile assemblies [164] in relation to the origin of life, as detailed in a chapter on amphiphile advantage in [53]. Most small hydrophilic organic molecules have negligible affinity towards each other, and if they show high mutual affinities, this would lead to sparingly soluble solid crystals or tar [12]. The remarkable advantage of amphiphiles is their strong mutual affinity, leading to a capacity to form fluid phases, distinct from the aqueous surrounding. This is because amphiphile mutual affinity is mostly due to the hydrophobic tail–tail interactions [90] and has relatively small dependence on the functional hydrophilic headgroup. This situation allows hydrophilic headgroups to be proximal to each other without hindering function. Lipid aggregates are also size-limited, being either small spheres (micelles) or two-dimensional sheets (bilayers), thus offering easy access to aqueous molecules and preventing excessive aggregation. Combinatorial proximities of headgroups may generate surface catalytic centres, and in this respect, amphiphile assembly surfaces are alternatives to the often invoked prebiotic mineral surface [165], but with considerably higher flexibility and potential diversity. In a related vein, heterogeneous micelles bear similarity to globular proteins, being of similar size, having hydrophobic interior and hydrophilic functional surface [55] with catalytic capacities (see next section).

We also briefly address the question of the abiotic origin of lipid-like amphiphiles, so as to be convinced about their feasibility in an origin scenario, including terrestrial syntheses and celestial delivery [55,166]. GARD is very permissive about the types of amphiphiles it accommodates (§3), so it is unnecessary to ask about early syntheses for specific molecular structures, including those found in present-day life, for some of which the abiotic synthesis is deemed difficult. The only requirement for GARD participation is facile entry into micelles or bilayers. This includes amphiphiles as well as mildly hydrophobic compounds, including, e.g. short-chain fatty acids. As the hydrophilic headgroup may assume almost any structure, its synthesis is covered by the run of the mill accounts on abiotic organic chemistry [167]. The availability of amphiphiles with sufficiently long hydrophobic tails is amply substantiated [168–171] as is that of hydrophobic and amphiphilic peptides [44,172]. Equally positive is evidence for vesicle-forming lipids in carbonaceous meteorites [173,174], bilayer-compatible polar hydrocarbons (complex mixture of ketones, *S*-heterocycles and *N*-heterocycles) [175] and generally of hydrophobic compounds in accretionary infall [166,176–178]. Finally, lingering doubts about whether abiotically synthesized short-chain lipids are sufficient for vesicle formation are alleviated by the demonstration that small amounts of long-chain lipids promote short-chain lipid vesicle formation [83].

Finally, it is important to refute a widespread notion about GARD. This model does not purely belong to the ‘compartment first’ school, because in its simplest embodiment, it is not about providing enclosures for other molecules. ‘Compartment first’ typically implies that lipids form an inert vesicular enclosure, and other molecules, with different chemistry (e.g. self-replicating or metabolites) use this vesicle for protection. By contrast, GARD entails a lipid phase (micelle or vesicle) that in itself is a diverse and dynamic chemical arena that embodies mutual catalysis and reproduction, that takes place in the lipid phase. In addition to amphiphiles, this could involve non-polar molecules in the hydrophobic tail regions of micelles. The latter situation is akin to that embodied in another lipid-based model for early evolution [179]. As conveyed, in detail, in earlier parts of this section, and in table 1, the lipid phase has numerous advantages in the context of life's origin, and under certain conditions, may actually seed life without the involvement of enclosed water-soluble molecules. This justifies the term ‘Lipid World’ for this scenario, where lipids are both amphiphilic and catalytic. Notably, the more advanced GARD version (M-GARD, §11.1), delineates steps towards a *bona fide* protocell, whereby lumenal soluble molecules *are* invoked. However, these hydrophilic molecules jointly succumb to the mutually catalytic dynamics of the GARD formalism, along with the lipids in the shell, meaning that the enclosing bilayer is still very far from merely constituting an inert compartment. Additional notes on the transition from lipid-centric early chemistry to more life-like chemistry are presented at the end of §11.2.

**Table 1.** Advantages of lipid assemblies.

form spontaneously from dilute solutions
fluidic: allow easy exchange
opportunistic: any lipid can take part
condensed organic phase: promotes interactions
may harbour compositional information
may manifest significant catalysis
undergo facile fission
may encompass aqueous volume: vesicles
afford evolutionary continuity towards protocells
heat stable: allowing early emergence

### 10.1. Lipid catalysis

Lipids are widely thought of as chemically inert non-catalytic compounds whose role is to form membranes. An important aspect of this misconception is the ‘like it is today’ outlook, which infers amphiphile chemical properties based on the molecule types found in present-day cells. However, as part of introducing the ‘Lipid World’ scenario [55], we reviewed the early evidence that catalysis is not restricted to proteins and RNA, and that lipids and other amphiphiles actually have considerable catalytic function. This was based in large part on the monograph by Fendler [180]. Facing the convincing evidence, we introduced the term ‘lipozyme’ to indicate a lipid molecule endowed with rate-enhancement capacities. This section is aimed at further elaboration of the catalytic capacities of lipid-like molecules. All cases described belong to the category of non-enzymatic (or enzyme mimetic) catalysis, as reviewed [47,181].

There are published examples in which lipids in membranes exert non-covalent catalysis. An interesting case, where both catalyst and catalysed molecules are lipids, shows that small concentrations of long-chain fatty acids enhance the rate of incorporation of short-chain fatty acid into vesicles [83]. Such a non-stoichiometric effect, described by the authors as cooperativity, hints at the possibility of lipid catalysis affecting another lipid joining an assembly. The same group also reported that a hydrophobic membrane-incorporating peptide can enhance fatty acid uptake into vesicles [182]. These cases lend direct credence to catalytic effects of the kind assumed in GARD, namely lipid-catalysed lipid joining reactions.

Other examples are those in which only the catalyst is a membrane amphiphile. A well-studied case is lipid catalysis of the non-covalent binding of ligands to membrane receptors [183,184]. In-depth kinetic analysis of this catalytic effect [185] points to general mechanisms such as reduced dimensionality, enhanced local ligand concentration and constrained ligand orientation. In parallel, a more molecularly specific mechanism is offered, which includes intermediate steps of ligand adsorption to the membrane, followed by ligand insertion into the membrane at specific binding sites before ligand–receptor docking. The first two steps are analogous to the proposed GARD mechanism of catalysed lipid entry. In an analogous system, clue to the catalytic mechanism was a demonstration that the peptide ligand underwent structural changes upon binding to micelles [184].

Another relevant case is the reported ×1000 acceleration of the nucleation of α-synuclein aggregation by lipid vesicles [186]. This is likely mediated by a lipid-induced catalytic effect on the folding of the α-synuclein protein [187]. In an additional relevant study [188], membrane lipids act as allosteric ligands for the peripheral membrane protein enzyme phospholipase A_2_. Upon binding of the allosteric ligand, the enzyme shifts its conformation to an open state, extracts another lipid molecule from the membrane and induces its hydrolysis. Here, the allosteric lipid may be thought of as a co-enzyme for the phospholipase protein enzyme.

Lipids have also been shown to catalyse covalent reactions. Such precedents are necessary to support the M-GARD model (§11.1). In two examples, lipid bilayers have been shown to promote the polycondensation of amino acids to peptides [189,190]. An analogous case is the oligomerization of thioglutamic acid, where lipid surfaces were reported as catalysts for peptide bond formation [191]. In two other cases [103,192], lipid-catalysed covalent bond cleavage led to the conversion of a lipid precursor to lipid, enhancing vesicle growth and division. In the latter case, the non-enzymatic catalyst was specifically identified as a rather simple, abiotically compatible molecule: an imidazole headgroup (the sidechain of histidine), linked to an 18-long hydrocarbon chain.

Finally, a highly pertinent case of one lipid catalysing the covalent modification of another has been reported by Devaraj and co-workers [193]. The model lipid catalyst has a headgroup constituting a hydrophilic chelator for Cu^1+^ ions and the hydrophobic moieties were three modified fatty acid chains. This amphiphilic molecule served as effective catalyst for the covalent bond formation between an alkyne-derivatized lysophospholipid and an azide-derivatized fatty acid analogue, leading to the formation of two-chain phospholipid analogue. All above-mentioned compounds reside within the same vesicular bilayer, and the lipid catalyst affects compositional remodelling of this membrane. Such a scenario is of high relevance to the M-GARD model as described in §11.1–11.2.

For the standard GARD model, the relevant examples are cases of lipid-catalysed non-covalent reactions. While the examples are convincing, the consideration of catalysis for non-covalent reactions is somewhat unorthodox. However, the free energy diagram of a non-covalent reaction is not different from that of a covalent one (figure 10). Both include a passage from reactant to product via a transition state characterized by an energy of activation [194]. Such an energy barrier may be lowered by intermolecular interactions, giving rise to catalytic rate enhancements. A pertinent biological example of a catalysed non-covalent reaction is solute passage through a membrane channel [195]. In GARD, the elements of the *β* matrix represent analogous rate-enhancement events, whereby the joint catalytic effect of several amphiphiles in an assembly enhance the rate of insertion of an extraneous lipid into the same assembly (figure 10).

The examples of covalent catalysis by lipids open new vistas regarding the M-GARD model (see §11.1). Beyond the concrete published examples, it should be underscored that every reported case of non-enzymatic covalent catalysis [181,196–198] points to a potential lipid catalyst for the same reaction. In an example, the dipeptide catalyst seryl-histidine has been shown to catalyse the formation of a covalent peptide bond, generating another dipeptide [182]. This peptide exemplifies the general phenomenon of catalytic small peptides [199]. Thus, the lipid derivative in which seryl-histidine is a headgroup, linked to any appropriate hydrocarbon tail, would also likely serve as an effective catalyst, perhaps even better than the soluble version, due to facilitated proximity and reduced dimensionality.

## 11. Metabolism first

Mutually catalytic networks are widely considered as synonymous with the ‘metabolism first’ scenario [121,200–202]. Dyson [8, p. 49] relates to this scenario as deeply connected to his own model. He then describes GARD as an instance of mutually catalytic networks having to do with ‘the evolution of molecular populations … in which some of the molecules catalyse the synthesis of others’, and adds that ‘Conditions (exist) under which populations can evolve to a high and self-sustaining level of catalytic organization’. In fact, the GARD model in its present form succeeds only partially in capturing the essence of such a metabolism first scenario. GARD does address the crucial point: how a form of metabolism, without informational self-replicating biopolymers, may portray a capacity to self-replicate. However, the spirit of metabolism rests in a network of catalysed *covalent* reactions, and these are missing in the basic version of our model. This section and the next address such paucity, and narrates past and future relevant research directions.

The question of whether a metabolic network can replicate has been amply addressed. Trivial criticism would be that metabolism requires protein enzymes, which can only come about via the translation of self-replicating biopolymers. An adequate solution is invoking prebiotic non-enzymatic catalysts [181,196–198], including cofactors (coenzymes), hypothesized to be early precursors of present-day enzymes [203–205].

But can one be convinced that a classical metabolic network, e.g. the tricaboxylic acid (TCA) cycle driven by non-enzymatic catalysts will actually generate copies of itself? A curious misconceived inference is: ‘the cycle is described as autocatalytic (since) each molecule of citric acid introduced into the cycle results, after a turn of the cycle, in the generation of two molecules of citric acid’ [206, p. 0005]. Self-replication is viewed here as pertaining to only one among several metabolites, and also ignores the replication of the catalysts. A remedy would require experiments showing capacities to run the entire TCA cycles with non-enzymatic catalysts, as described [207], but include also the replication of the non-enzymatic catalysts. Also needed is a more formal approach to relate metabolism to established mutually catalytic networks formalisms or to ‘supernetwork’ computations as described for the specific case of the TCA cycle [197].

It is necessary to address some additional commonly expressed criticisms against the metabolism first scenario, in general, and against GARD as one of its examples. It is somewhat surprising that a literature search for reviews addressing ‘metabolism first’ in the last three decades retrieves practically only negative critiques. A representative instance is a review by Anet [26, p. 656], with points as delineated and addressed hereby:

(1) ‘Another elaboration of Dyson's model has been carried out by Lancet and co-workers, who have also considered that lipids might be present and form vesicles that act as cell-like compartments'. This is incorrect: GARD is not about compartments, but about a lipid-embodied replicating metabolism, as is clearly explained in §10 and in its quoted publications.
(2) ‘The treatment is abstract and mathematical without consideration of the properties of real molecules’. This is wrong: in using rigorous chemical kinetic equations, GARD is much less abstract than many other models for life's origin. Further, it is the only model in its genre that uses experiment-based graded catalytic parameters (§4) and in two papers also the properties of real lipid molecules [60,119].
(3) ‘These authors discuss the growth and subsequent cleavage of their “cells” and call the process “compositional replication”, but this nomenclature is confusing, as what happens is completely different from the replication of RNA or DNA’. This is a total misunderstanding: GARD's compositional information is rigorously defined and shown to be widely involved in present-day life (§5.2). Being based on a different information type does not make compositional replication faulty, and in fact opens new avenues for understanding information transmission before the advent of polynucleotides.
(4) ‘A compositional replication system has an inheritance that transmits a tiny amount of information compared with a polymer having unlimited inheritance, such as RNA’. Mostly wrong: it is indisputable that polymer sequences do better than assembly compositions. But the overwhelming difficulties for RNA emergence and replication in a prebiotic scenario make it a necessity to explore other models, where compositional information is natural. Then, as long as compositional replicators have *some* inheritance and *some* capacity to evolve, they become legitimate candidates for jump-starting life (§7).
(5) ‘Even Dyson has remarked that Lancet's computer-based treatment lacks the proper chemical information input to be useful’. Dyson [8] has been misread: the text relevant to GARD is ‘The simulations of the Oparin theory summarized by Lancet are a good beginning, but they still have far to go’. Having far to go is common to all origin of life models. The next sentence ‘None of the models incorporates enough details of the chemistry to provide a realistic test of the theory’ is a general statement that applies to *all* origin of life models.
(6) ‘Basically, the computer models (and even the back-of-the-envelope models) can be reasonable, but still give useless results because the data supplied to the model is faulty’. This turns the scientific method upside down. Models do not get falsified by faulty data but rather by correct data, and only if the model's predictions based on good data are incompatible with observations.

A more general critique on GARD has been put forth by Lazcano [25, p. 70], quoting Anet's paper: ‘There are no empirical indications that the autocatalytic metabolic first schemes that have been proposed could have self-assembled in the prebiotic environment’. This unwavering statement is unwarranted. First, because it is based on Anet's paper with its glaring fallacies, as delineated above. Second, because the alternative option, self-replicating RNA, does not fare better than catalytic networks regarding empirical evidence for spontaneous self-assembly in a prebiotic environment [6].

Thus, what would fare origin of life scrutiny best is to analyse the advantages and paucities of both competing scenarios, using objective and rigorous comparative implements. This may lead to useful syntheses towards a true decipherment of how life began.

### 11.1. Metabolic GARD

GARD fully embodies two widely accepted cornerstones of life's origin. The first, *compartmentalization,* comes naturally, as the model is built upon lipid micelles and vesicles. The second, *information storage/copying*, shows up in the across-generation propagation of assembly composition. However, the third cornerstone, *metabolism*, is currently only partially modelled in GARD, by including a network of mutually catalysed entry–exit non-covalent reactions. Non-covalent reactions are *bona fide* participants in modern metabolism, exemplified by ligand binding, protein folding, conformational transitions, solute translocation across membranes and more. However, it is obvious that for full analogy to present-day metabolism, GARD has to become compatible with covalent reactions.

We have already taken important steps towards this goal in several past publications. In two early papers [55,208], we have elaborated the concept that given appropriate kinetic model modifications in mutually catalytic GARD assemblies of monomeric amphiphiles, dimers and higher oligomers would form. These could replace some of the monomers, assuming their catalytic roles in the network. It was suggested that rearrangements of monomers within the oligomers would make the new assemblies more successful in propagating their compositions. Oligomers could also exhibit statistically higher catalytic potencies because of a combinatorial library effect [55,209]. In preliminary simulations, a defined size of monomer alphabet was reached and a hierarchy of oligomer sequences established [208]. In this paper, we noted that the large diversity of the molecular components thus generated would enhance the capacity of a GARD assembly to embody an unlimited hereditary potential as defined [210].

**Figure 11.**
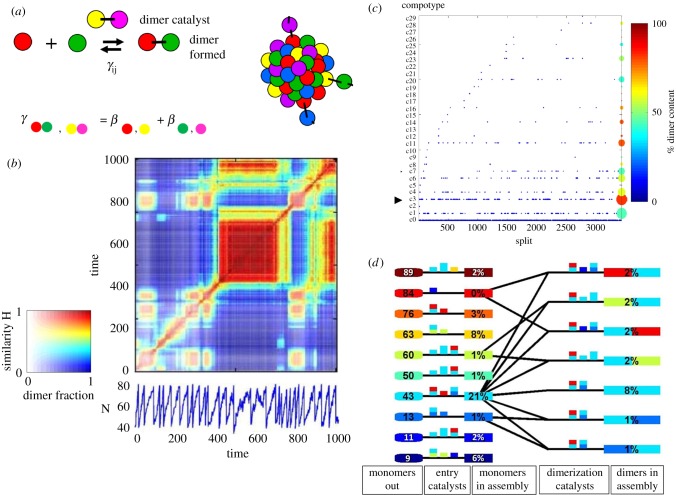
Polymer GARD (P-GARD) simulations, reaching up to dimers. All figures are modified from [57]. (*a*) A cartoon depicting reactions and catalytic processes considered by dimer-GARD. Coloured spheres represent different monomeric compounds *A_i_*. The dimerization reaction may be catalysed by both monomers and dimers. When a dimer is the catalyst as shown, the reaction proceeds by way of templating-like catalysis. The dimer-effected rate enhancement *γ_ij_* is obtained by on-the-fly computing (rather than matrix lookup), employing a combinatorial algorithm, reflecting an additive effect of catalytic sub-sites. (*b*) A compositional similarity diagram, analogous to that in figure 3*d*. Dimer fractions are indicated by the colour vividness (inset). The saw-tooth graph representing growth–split cycles depicts the size of the assembly in terms of molecular count (*N*) as a function of time (define as in figure 10), splits occurring at *N*_MAX_ = 80. The simulation is in trace mode as defined in figure 2*b*. Other parameters are as in [54]. In this simulation, a strong dimer takeover is observed between time points 400 and 700, suggesting that dimers become part of the composome and its replication dynamics. (*c*) Compotypes appearing in a dimer-GARD simulation run. The abscissa indicates consecutive splits. The identifiers C1–C29 on the left ordinate identify the 29 different compotypes, while C0 is drift. The dots in the body of the figure are individual appearances of composomes belonging to a given compotype. Each compotype is characterized by a circle on the right ordinate whose size reflects frequency of the composome's appearance and colour (as per scale shown) indicates the dimer content. (*d*) A schematic of a metabolism-like mutually catalytic network that underlies compotype C3 in panel *c*. Monomers identity is shown by arbitrary colours. The externally available monomers (ovals, monomers out) are marked by their serial repertoire identifier, while the monomers within the assembly (rectangle, monomers in assembly) are labelled by their % propensity within the assembly. The catalytically formed covalent dimers are also labelled by their % propensity (rectangle, dimers in assembly), and are identified by the two colours of their constituent monomers. Two types of catalysed reactions are: entry/exit of monomers from the environment into the assembly (entry catalysts) and covalent bond formation/break within the assembly (dimerization catalysts). For each of these reactions, their catalysts (monomers or dimers) are shown above the reaction line, with identities disclosed by the colour code. Some of the monomers (9, 11 and 89) undergo catalysed entry but do not take part in dimerization reactions.

In a later paper [57] we presented polymer GARD (P-GARD), an extension of the basic GARD monomer-only model (figure 11). Most of the analyses were done on a simple case, limited to dimers only. Basic GARD, with its *β* matrix, served as a replication infrastructure for catalysed oligomer formation. This was done using nature-mimicking recognition rules, akin to string matching, to compute on the fly the catalytic parameters for the formation of oligomers and their own catalytic potencies (figure 11*a*). Thus, dimers were formed internally, marking a transition for GARD from pure heterotrophy to partial autotrophy. We observed events of ‘dimer takeover’—the emergence of composomes with appreciable dimer content (figure 11*b*,*c*). It was possible to view the mutually catalytic networks in detail, witnessing how both monomer and dimers may catalyse the entry–exit reactions as well as the formation and breakdown of dimers (figure 11*d*). A simulation under constant population conditions showed the dynamics of takeover and extinction of dimer-containing composomes [58]. These results highlight an important principle: that reproduction based on homeostatic growth is apparent not only in a simplistic join–leave setting but also in a scenario of covalent bond making and breaking, constituting an example of self-replicating biosynthetic metabolism. This is echoed in a similar model for an oil droplets system with catalysed covalent modifications [32].

Based on this initial work, we are now developing a new version of GARD simulations under the title M-GARD [211]. This is aimed to go beyond the mere capacity to accommodate covalent chemistry, striving to introduce features that faithfully mimic present-day cellular metabolic functions. All such simulations are based on the GARD's compositional replication/reproduction doctrine. This includes time-dependent compound concentrations governed by kinetic equations, with rate constants and catalytic rate-enhancement factors that span a graded true-to-experiments scale. We plan to rely on standard biological scrutiny modes, exemplified by metabolic flux analyses that depict how unicellular organism grows homeostatically upon absorbing specific food compounds [212]. An appropriate compromise will be kept between attributes that are entered *ad hoc* and those that constitute emergent phenomena, as exemplified [68].

M-GARD posits that the vesicle membrane components should have a large diversity of hydrophilic headgroups, which could take part in mutually catalytic metabolism on the lipid bilayer surface. The molecular diversity, effectively constituting an augmentation of the GARD repertoire *N*_G_, would be partly the result of a higher diversity exogenous entry, but in addition would stem from endogenously catalysed syntheses. As evidenced by the above-mentioned P-GARD simulations, such internally synthesized compounds will take part in GARD composome dynamics, i.e. get propagated from one generation to another. This means that if a good catalyst is mutually catalytically synthesized, it will persist along generations, solving the catalyst replication problem pointed to in the context of replicating metabolism (previous section).

Just as basic GARD's compositional reproduction and inheritance make it possible to temporarily forgo polynucleotides as information carriers, it is possible for M-GARD to make do without protein enzymes. In an extension of Kauffman's peptide-based formalism, M-GARD will use low molecular weight lipid headgroups as non-protein catalysts for the covalent formation or modification of other lipids. This notion obtains further support from the experiment-based proposals that present-day low molecular weight cofactors may have been early non-protein catalysts [32,204,205,213,214]. Internally catalysed synthetic reactions could enrich the membrane with new compounds with new catalytic properties.

Simulated M-GARD chemical reactions, each modelled by kinetic equations, are slated to take place on both the outer and inner leaflets of a simulated vesicle membrane. Among such covalent transitions would be headgroup extension and clipping. If extension occurs on the outer leaflet and following leaflet flipping [215], and clipping takes place on the inside, this would be effectively equivalent to catalysed transport (figure 13*a*). On occasions that the extension reaction involves an exogenous high-energy precursor this might amount to rudimentary active transport [47,59]. Finally, if, prior to clipping, endogenous covalent modification occurs on either the outer or the inner leaflet, the lumen will become populated with a *de novo* molecule, not present in the environment. Both of the last-mentioned reactions point to the possibility that an M-GARD vesicle will gradually build an idiosyncratic lumenal content, different from that of the external milieu. Such reactions, if occurring repeatedly, can generate longer membrane attached or lumenal (soluble) oligomers. This far-reaching scenario suggests the possibility that the membrane would serve as a catalytic arena for synthesizing lumenal molecules, including polymers. This is the inverse of the often invoked scenario, where lumenal catalysts generate membrane-forming compounds [36,66].

Many of the above-mentioned reactions obviously require a free energy supply to afford facile (energetically downhill) syntheses. This is in line with GARD being an obligatory away-from-equilibrium system. As discussed, what drives non-covalent GARD are lipid hydrophobic tails, whose mutual association is energetically favourable. But for covalent syntheses, it is necessary to faithfully simulate other free energy resources. This could happen via external feeding by exogenous (abiotically generated) high-energy headgroup modifiers, e.g. pyrophosphate derivatives [217], or thioesters [218], and even high-energy lipids such as polyprenyl phosphate [170]. Relevant to this is the demonstrated trans-thio-esterification that leads to spontaneous non-catalysed lipid remodelling [219]. This example points out the potential contribution of non-catalysed reactions involving high-energy precursors in mutually catalytic networks. Indeed this option is modelled in some RAF versions that invoke a rate constant for the spontaneous uncatalysed reactions [216]. In GARD, spontaneous reactions belong to the basic infrastructure, including rate constants for the forward and backward uncatalysed reactions (figure 1).

In the long run, autonomous protocells cannot rely solely on being fed high-energy compounds, presumably generated based on thermal energy [220]. The most likely alternative energy source is light. Photons (including in the visible spectrum) are known to exert chelated metal-mediated photoredox catalysis, leading to small organic molecule activation [221]. This line of thought is supported by ascribing a central role to light energy in life's origin [104,222] and by an experimental utilization of a photoredox reaction in an origin of life context [223]. Photochemistry can be easily added to the kinetic equations of M-GARD. We note that in this respect M-GARD is slated to merge two central origin of life views [224, p. 737] between ‘the progressive complexity of organic matter described by Oparin (which) argued for a heterotrophic process' and ‘Haldane, taking a contrary stance … (that) insisted on the role of light in chemical synthesis, and preferred an autotrophic process’.

The M-GARD model described herein strongly echoes a portrayal by Pohorille and co-workers [150, p. 357] on the possible coevolution of membrane components with metabolism. This is described as ‘continuous, evolutionary path that connects nascent biochemistry with simple, membrane-bound oligopeptides, ion channels and … membrane proteins capable of energy transduction and utilization of energy for active transport’. Our M-GARD model is also similar in general outline to that published by Tessera [161, 162, p. 559, 163], whereby ‘vesicles with bilayer membranes … are able to self-reproduce’ and then with a ‘plausible scenario for the emergence of a positive feedback process … (assume a) capability of evolving’. Some added value of M-GARD is its capacity for quantitative computer kinetic analyses, allowing verification of homeostatic reproduction of entire assemblies, including all their molecular components and enclosed lumenal content, so as to enhance the similarity to typical protocells.

### 11.2. Metabolic GARD experiment

**Figure 12.**
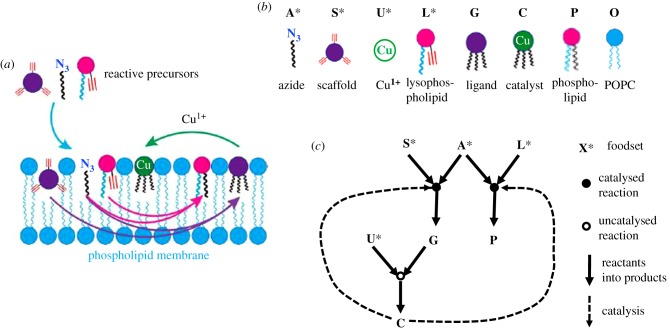
(*a*) Summary diagram of a study of self-reproducing catalyst that drives recurred phospholipid synthesis and membrane growth [193]. The experiment starts with vesicles of a regular phospholipid (palmitoyl-oleoyl-phosphatidyl choline, POPC), shown in light blue. Figure reproduced from [193]. (*b*) As also shown in *a*, other components added to the reaction vessel are: 1-azidododecane (azide), tripropargylamine (alkyne scaffold), Cu^+1^ cuprous ions, alkyne modified lysolipid and Tris-(lauryl triazole)amine (TLTA, ligand). Then, in a spontaneous uncatalysed reaction, the TLTA ligand binds Cu^+1^ ions to form some copper-chelated oligotriazole (catalyst). This membrane-embedded lipid catalyst is capable of enhancing the rate of two reactions within the bilayer. The first, catalytically generates phospholipid from lysolipid and azide, and the second, catalytically generates more lipid catalyst. This self-reproducing catalyst drives triazole phospholipid synthesis and membrane growth. The carrier lipid palmitoyl-oleoyl-phosphocholine (POPC) does not take part in the reaction network. (*c*) The above chemical reactions are presented in a mutually catalytic network diagram format [216]. Four food compounds (labelled by asterisk) participate in two catalysed and one uncatalysed reaction. The scheme is formally not reflexively autocatalytic (RA) [216] because one reaction is uncatalysed. Yet, because that reaction occurs spontaneously with good efficacy, the system appears to show catalytic closure. Further, as described in the text, after many serial transfers, the carrier lipid POPC gets diluted out and this network becomes capable of showing catalysed homeostatic growth (preservation of the ratios of all components).

After further analysis, our interpretation of the article by Devaraj on lipid-catalysed lipid modification [193] (figure 12) leads to a rewarding conclusion. The experimental results provided are describable by a simple mutually catalytic network in line with the Kauffman–Hordijk model, as well as by the formalisms of M-GARD (previous section). The lipid-embodied network has four food compounds, three of which generate a lipid catalyst in two consecutive reactions. The catalyst then enhances the rate of its own formation, as well as that of a reaction generating a two-chain phospholipid from a single chain phospholipid and a fatty acid analogue. The ensuing network (figure 12*c*) does not strictly conform to the basic RAF definition as one of the reactions is uncatalysed, but it does conform to modified RAF criteria that accommodates spontaneous (uncatalysed) reactions [225].

Can a vesicle as described in this study faithfully replicate its entire composition? The network structure alone does not guarantee that, as homeostatic growth depends also on the inter-relations among all the kinetic parameters and molecular concentrations. Also, initially the vesicles contain large amounts of POPC, lipid that does not take part in the network. But it turns out that at the end of a serial transfer procedure employed, this initiator non-network lipid is diluted and the vesicles remain only with the components of the network as shown in figure 12*c*. It thus appears that the mutually catalytic vesicles asymptotically reach the state of possible consistency with an M-GARD composome with homeostatic growth. Future experimentation and simulations with the appropriate concentrations and kinetic parameters should be used to further substantiate this claim, and verify that the described results indeed constitute a first experimental enactment of a GARD composome.

This merger of experiments with computer simulations should show the way to future developments. M-GARD will in all probability fulfil the expectation for a capacity to evolve, as its replication attributes are identical to those of the basic GARD model. Its design embodies prospects for gradual progress towards greater molecular complexity and catalytic capacities, similar to such progression in Kauffman's model. Along with that, come stronger and more selective interactions, via a relationship between affinity and selectivity known to exist in ligand–target interactions in general [65,226].

**Figure 13.**
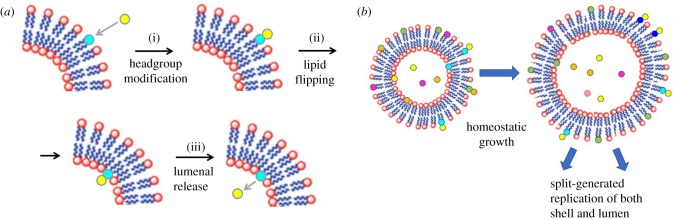
Example features of the Metabolic GARD (M-GARD) model. (*a*) The reactions shown are assumed to be non-enzymatically catalysed by other lipids as part of a GARD mutually catalytic network, analogous to that of the standard GARD model. (i) Lipid headgroup is modified on the outer leaflet of the bilayer, via a covalent reaction with an extraneous hydrophilic compound (yellow); (ii) non-covalent lipid flipping to the inner leaflet; (iii) covalent clipping that releases the hydrophilic compound into the vesicle aqueous lumen. This progression is equivalent to catalysed transport that confers chemical specificity on lumen enrichment. In modified form, if a high-energy lipid (e.g. with oligophosphate headgroup) is involved as a reactant, this could constitute a primitive form of active transport. (*b*) The reactions in *a* could underlie a coveted scenario in which under modified GARD compositional reproduction dynamics homeostatic growth and fission could lead to the generation of progeny with faithful copying of the composition of both the bilayer shell and the enclosed aqueous lumen.

M-GARD is slated to provide a useful platform for future detailed inquiry, joining simulations with experiments. In the future, it could at least partly represent further evolutionary improvements, portraying increasingly long polypeptides, by a polyketide-like consecutive amino acid addition [227]. Perhaps even show the way to templating oligonucleotides and very primitive translation-like paths. Another future challenge will be to demonstrate that M-GARD assemblies reveal replicating composomes that encompass all its chemical capacities, including the enclosed lumenal content (figure 13*b*). Reaching these milestones will allow M-GARD to become a workable route towards a much more life-like protocell.

A question often asked is how lipid-based GARD might lead to oligonucleotide chemistry, which constitutes the basis for templating informational biopolymers. In other words, how could reproducing composomes discover the new laws of strand complementarity? As lucidly pointed out by Danchin [228], molecular complementarity is a much broader phenomenon than polynucleotide strand recognition and is seen, for example, in the self-reproduction of the tubulin structures of the centriole. Likewise, at the basis of GARD assembly reproduction are diverse events of inter-lipid molecular complementarity, mediated by chemically diverse headgroups, and underlying mutual catalysis. A lipid-based path to the specific emergence of nucleotide-mediated complementarity could involve nucleotide lipids, as reported in certain bacteria [229]. Such nucleotide lipids could be absorbed from the environment, where they might be formed by abiotic reactions or reaction networks. Some other headgroups, including certain nucleosides and nucleotides could be synthesized via endogenously catalysed headgroup modification reactions such as are shown in figure 13*a*. A tremendous advantage of this endogenous metabolic synthesis in an M-GARD assembly is that certain synthetic capacities will be heritable by the GARD reproduction mechanism, thus representing the emergence of partial autotrophy, applying to certain compounds.

In the M-GARD framework, nucleotide lipids could further undergo successive covalent modifications to become oligonucleotide lipids, and such could interact within lipid bilayers via strand complementarity to direct the synthesis of oligonucleotides on adjacent lipid molecules. These and similar molecular events, occurring in a compositionally reproducing lipid assembly, could serve as very primitive arenas for further burgeoning of evolving oligonucleotide chemistry, including lumenal soluble oligonucleotides, constituting rudimentary strand replication within M-GARD.

The foregoing description of a transition from lipid chemistry to oligonucleotide chemistry is reminiscent of an evolutionary takeover proposed by Cairns-Smith [230], transitioning from early replicating mineral clay chemistry to that of polynucleotides. Of note, such a transition is much more profound than that starting with lipids, because it involves a very different set of elements such as silicon and aluminium, whereby information is held in the form of aluminium for silicon lattice substitutions. This makes the takeover to life as-we-know-it much less straightforward. Such a mineral-involving model is different from that proposed by Baum [29], whereby mineral faces foster the emergence of surface-associated organic chemical consortia capable of adaptive evolution. Since here the evolving entity is organic, takeover is likely to occur much more readily. Consequently, it is likely that in the clay model what we would witness today is a palimpsest [231], with meagre traces of the ancient past, while for the other two models we could possibly observe archived relics of earlier evolution.

### 12. Systems protobiology

**Figure 14.**
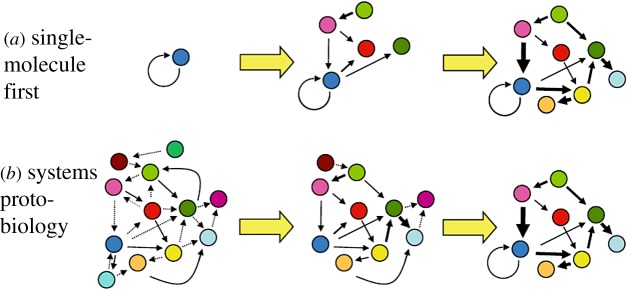
Two alternative views of the progression from early molecular entities to more life-like networks. (*a*) Early evolutionary steps involve one molecule type with template self-replication capacity, recruiting other components and progressing gradually towards the evolutionary target by increasing network size. (*b*) Early networks with a large number of nodes and low interaction fidelity progress gradually by node weeding and modification, undergoing fidelity enhancement, towards the same evolutionary target. Reproduced from [85].

It is widely accepted that somewhere along the line towards the LUCA, a web of mutually interacting molecules must have emerged [24], as prevalent in all present-day cellular life. Yet, investigators are divided on the time point at which such web first appeared. According to the RNA-first scenario, the first protobiotic entities constituted one or very few types of (self-replicating) molecules, and molecular networks came later (figure 14*a*). In this line of thought, the networks are often thought to have emerged separately, as metabolism, and then be somehow joining forces with the replicators. This must have been done in a cunning way that made the preformed networks become instructed by the separately long-existing replicating polymers.

By contrast, the mutually catalytic network view asserts that protolife was networks from day 1, in the spirit of systems protobiology [91] (figure 14*b*). This view asserts that the first to arrive upon the scene were spontaneously forming assemblies that included networks of simple organic molecules, belonging to ‘Monomer World’ [102], which had capacities to interact catalytically with each other. The RAF and GARD formalisms point to the realistic possibility that some of these networks were endowed with a capacity to make copies of themselves, perhaps not much less chemically improbable than non-enzymatic replication of unaccompanied RNA. Such early networks could gradually become more efficient in terms of mutual catalysis fidelity, and hence reproduction capacity (figure 14*b*). Being both networks and replicators, they could avoid the lower likelihood of the replicator–metabolism force-joining event.

There is a basic physico-chemical advantage to the catalytic networks scenario relative to the RNA-first scenario, related to the latter's single molecule type characteristic. It is clear that the chemistry of early earth was extremely heterogeneous. In addition, the chemistry of living cells today is very far from a single molecule type simplicity. A mutually catalytic networks origin is, therefore, much more in line with a continuity principle of life's origin, prescribing that ‘from any stage in biogenesis, a continuous series of plausible transitions must lead backward in time to the non-living geochemistry of the planet … (This) puts the burden of proof on any theory that requires discontinuities’ [104, p. 282]. Accordingly, postulating that a huge abiotic compound repertoire passed through a single molecule (RNA), only to go back to high diversity carries a dear discontinuity price.

The exploration of mutually catalytic networks comes with an important methodological advantage. Students of mutually catalytic networks basically operate in a realm describable as ‘systems protobiology’, and, therefore, can analyse their models using the toolboxes of systems biology [232,233] and systems chemistry [7,23]. This is exemplified by our GARD analyses as described [85,91]. At the same time, such early systems view has promise for merging the two conflicting origin of life scenarios (autocatalytic networks and RNA world), as pointed out by Luisi [234, p. 1]: ‘Another new wind in our field comes, in my opinion, from the development of system biology … . It is perhaps because of this new thinking that the two main “parties” on the origin of life … are coming more and more close in contact’. Finally, Mann, with a goal of seeking ‘key life criteria required for the development of protobiological systems' [235, p. 2136] has written : ‘It therefore seems reasonable to propose that one of the key steps in the formation of a hypothetical protocell involved the spontaneous self-ordering of a mixture of abiogenic molecules under appropriate conditions into compartmentalization modules capable of primitive forms of replication or metabolism, or both. This notion constitutes the basis of bottom-up approaches to the laboratory construction of protocell models exhibiting minimal representations of the core criteria of life’.

This section would not be complete without relating to Lazcano's poignant question regarding life's origin: ‘Is a System-Level Understanding Feasible?’ [25, p. 73]. The answer provided in his paper is definitely negative: ‘As of today, theoretical models of self-organized complex metabolic systems have not led to radical changes in current concepts of heredity and evolution, nor have they provided manageable descriptions of the origin of life. In some cases invocations to spontaneous generation appear to be lurking behind appeals to undefined “emergent properties” or “self-organizing principles”, that are used as the basis for what many life scientists see as grand, sweeping generalizations with little relationship to actual biological phenomena. In spite of many published speculations, everything in biology indicates that life could have not evolved in the absence of an intracellular genetic apparatus able to store, express, and, upon replication, transmit to its progeny information capable of undergoing evolutionary change’.

Answers to every one of these allegations are provided in the present review. Briefly:

(1) Complex metabolic systems are not more ‘theoretical models’ than the quasi-species model, which conjecturally assumes the existence of unaided self-replicating RNA.
(2) Mutually catalytic networks provide more ‘manageable descriptions of the origin of life’ than RNA world, in invoking detailed chemical kinetics equations testable both by experiment and by rigorous computer simulations.
(3) Prebiotic spontaneously forming replicating RNA fares much higher on the scale of ‘invocations to spontaneous generation’ than networks composed of easily available prebiotic molecules.
(4) Calling emergent properties and self-organizing principles ‘grand, sweeping generalizations with little relationship to actual biological phenomena’ is stark incongruity, considering that PubMed searches show 1390 results for ‘emergent property(ies)’, and 7720 for ‘self-organization(nizing)’.
(5) Stating that ‘Everything in biology indicates that life could have not evolved in the absence of an intracellular genetic apparatus' reflects the worst stumbling block to deciphering life's origin: the unwarranted assumption that life's beginnings must have reflected present-day molecular devices. The alternative stand is supported, inter-alia, by statements such as ‘it certainly seems more plausible that guided translation arose via a pre-existing selective process than that it arose spontaneously without prior adaptive evolution’ [29, p. 483].

## 13. GARD at planetary scale

As a preamble, we should revisit the definition, ‘Life is a self-sustaining chemical system capable of Darwinian evolution’. Similar definitions hold that ‘Life is that which replicates and evolves' [236, p. 14924] and ‘Life entails autonomy and open-ended evolution’ [1, p. 330]. Thus, the widely used term ‘prebiotic evolution’ is an oxymoron. For clarity, we propose to use ‘abiotic’ and ‘prebiotic’ to address different stages, perhaps increasing complexity of the chemical processes that took place before the advent of replication and evolution. The term ‘protobiotic’ (reflecting ‘protocells’) would then describe entities that show even rudimentary replication and Darwinian evolution, hence positioned across the line between non-life and life. Thus, by definition, the two competing scenarios for life's emergence, ‘RNA-first’ and ‘catalytic networks first’, belong to the realm of protobiology.

The origin of life should be regarded as a planetary phenomenon, unless clear evidence is available to the contrary. This is true despite Darwin's often quoted aphorism regarding life's origin in a ‘warm little pond’ [237]. Many scenarios still invoke specific environmental niches, as exemplified by hydrothermal suboceanic vents with high temperature and pressure [159]. But relevant to this example, we note that similar conditions may have prevailed on the entire early planet with predicted ocean temperature of 230°C under a 215 bars of CO_2_ atmosphere [238]. Such high temperatures, irrespective of site, would indeed promote certain prebiotic processes, including certain organic syntheses as well as amphiphilic assembly formation, the latter because high temperatures strengthen hydrophobic interactions [239].

**Figure 15.**
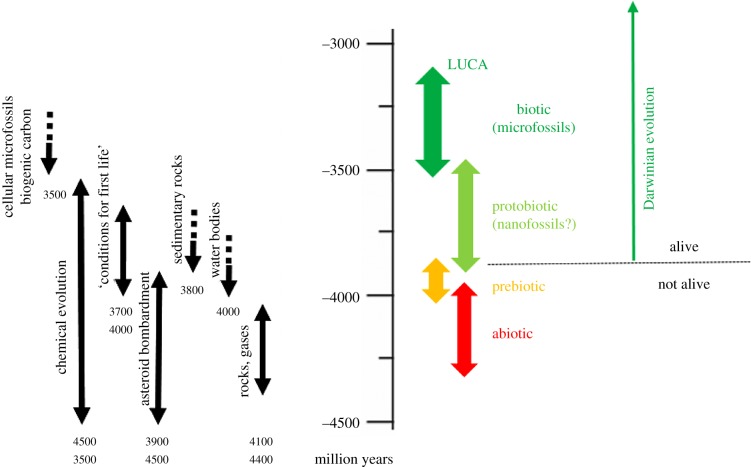
Origin of life as a planetary phenomenon. The geological timing data on the left are from [237]. The abiotic to biotic progression is portrayed with speculative and somewhat arbitrary time demarcations, with some justifications as detailed in the text. The not-alive/alive demarcation is between prebiological and protobiological entities, defined by the emergence of replication and evolution [2]. We note that a different but similarly idiosyncratic demarcation has been proposed [240]. The distinction between ‘abiotic’ and ‘prebiotic’ remains obscure, as indicated by having both appear interchangeably in a paper entitled ‘Abiotic synthesis of RNA in water: a common goal of prebiotic chemistry’ [241]. A possible distinction could be the degree of chemical elaboration: abiotic might relate to non-biological chemical processes that convert inorganic compounds or very simple organic compounds to somewhat more complex organic molecules, while prebiotic possibly more related to non-biological processes involving higher non-covalent or covalent complexity, sometimes necessitating external catalysis. The terms protobiotic, as exemplified in §12, addresses entities such as replicating RNA molecules or mutually catalytic sets, capable of rudimentary self-replication. More elaborate, membrane-enclosed replicating entities that are sufficiently large and durable to be detected as cell-like microfossils are termed here as biotic. We note that future technologies would perhaps be able to identify the much simpler and smaller protobiological ‘nanofossils’. Early biotic entities would likely be very different from LUCA, the last universal common ancestor of today's biota. Reaching this ultimate milestone has been described as ‘a long long way to LUCA’ [240, p. 4]. Every step in the passage from early protobiotic entities must have occurred, by definition, through Darwinian evolution.

To help comprehend the planetary facets of life's origin, it is useful to tentatively tie the abiotic to biotic progression with the early geology and palaeontology timetable (figure 15). Abiotic and prebiotic reactions are tentatively shown as occurring at the two sides of the Earth's hydration. For the more recent events, we offer a functional definition whereby the boundary between protobiotic and biotic processes coincides with the time at which cellular micrometre-size fossils appear 3.5 billion years ago [242]. This reflects the idea that early replicating entities, such as mutually catalytic sets or self-replicating RNA molecules, are not expected to generate fossils discoverable by current technologies (figure 15, legend).

Two key features in figure 15 are the short time assigned to prebiology (non-replicating) and the long time ascribed to protobiology (early replicators). The transition between the two is the appearance of NASA-defined life. We propose that this has happened relatively early based on inferences derived from GARD dynamics, showing that primitive self-reproduction capacity could be relatively easy to attain (see below).

**Figure 16.**
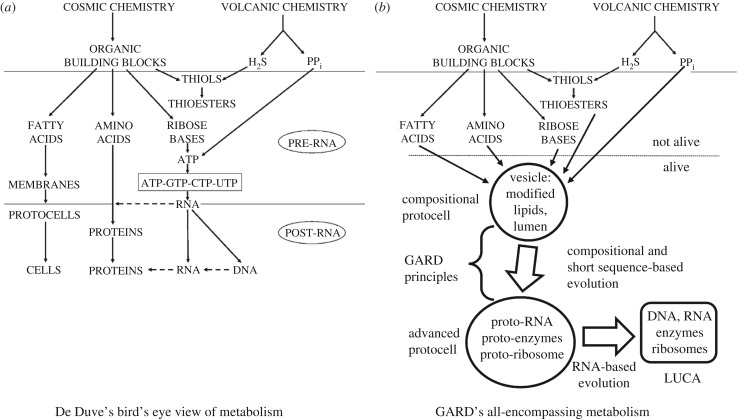
(*a*) Bird's eye view of protometabolism leading to life, published as tribute to Leslie Orgel by De Duve [217]. The scheme proposed conforms to the notion that the early chemistry of life, or protometabolism, must have prefigured present-day and GARD's System metabolism. The top part illustrates that two fundamental ingredients of life, inorganic pyrophosphate and hydrogen sulfide, both key energy transactions of present life, are likely to arise spontaneously in volcanic surroundings. Despite text descriptions of ATP as occupying a central position in all facets of protometabolism, the figure only shows its role in the advent of RNA. The scheme depicts protolife as involving three hardly interacting threads, with amino acids leading to proteins and fatty acids generating membranes. Disappointingly, protocells and cells are shown as an outcome of lipid membrane formation, without any shown relationship to the other two threads. Still, in line with what leads to the alternative scenario shown in *b*, De Duve states: ‘The early catalysts … cannot possibly have displayed the exquisite specificity of present-day enzymes and must necessarily have produced some sort of “dirty gemisch” … In particular, it is most unlikely that protometabolism could have happened by chance to generate just the two canonical pairs of complementary nucleotides. Such a fortunate coincidence smacks too much of prescience’. (*b*) In GARD's framework, the prelife period (not-alive) involves a plethora of abiotic paths that populate the planet with a very large assortment of compounds, including, but definitely not limited to the ones shown. We regard the separation of three threads in *a* as much less likely to have led to integrated protocells, precursors of the last universal common ancestor (LUCA). Instead, GARD and M-GARD paint a profoundly different picture, that of systems integration of some or all abiotic compounds into integral entities, that by the demonstrated GARD dynamics are capable of self-replication and rudimentary evolution, hence are alive by NASA's definition. The initial GARD assemblies would mostly just absorb amphiphiles from the environment and undergo primitive compositional replication. However, GARD's chemical opportunism would allow compositional protocells to emerge that include catalytic and high-energy amphiphiles to generate a replicating protometabolism as described under the M-GARD title. In a very long series of evolutionary events, but in principle kinetically traceable, complexity would increases towards producing primitive versions of templating oligomers (proto RNA [243]), as well as protein-based protoenzymes and even proto-ribosomes [244,245]. This scenario would make it much less necessary for early life to depend on the less efficient ribozyme catalysis. Additional eons of evolution would lead to LUCA, with full-fledged cellular machinery that will eventually win numerous selection bottleneck battles, becoming dominant in further evolution.

We depict the protobiotic period as especially lengthy, echoing the sentiment that the passage from the first molecular replicators to advanced (but non-LUCA) cells with DNA-like replication, elaborate metabolism, functionalized membranes, protein-like enzymes, RNA-like encoding and proto-ribosomal translation (figure 16) is likely to be extremely lengthy. A similar time span may be required for the evolution to further hone their molecular contrivance and reach the LUCA finish line. The spirit of these arguments has been captured by the statement: ‘Authors of review articles and textbooks are not always immune to streamlining the arduous pathways from molecules to cells' [237, p. 1266]. If such a timescale is correct, it appears that time to LUCA was in the range of 1 billion years, comparable with 1 billion additional years before eukaryotes emerged and another 1.5 billion before the dawn of multicellular organisms.

### 13.1. Life's probability

It is interesting to ponder the impact of the vast volume available for early chemistry and protobiology. If we consider only 1/50 of total earth surface area, i.e. 1 × 10^7^ km^2^, and if only the top 1 m of the aqueous surface is counted, we get a volume of 1 × 10^14^ m^3^. If a replicator with a diameter of 10 nm (roughly representative of a micelle with approximately 100 lipid molecules) is present in a pessimistically dilute suspension of 10^−5^ by volume (computed for a lipid with molecular mass of 500 Da, this amounts to a 20 µM concentration), one replicator is present a volume of 10^−19^ m^3^. This means that the above-mentioned planetary volume can contain 10^33^ (decillion) replicator instances (see also [59]). Gratifyingly, the use of this number allows the GARD model to generate important insights regarding life's emergence and probability, helping to indicate whether life might be a cosmic imperative [246]. The computations shown below are meant as an example, admitting that the numbers do not necessarily reflect realistic prebiotic scenarios.

Consider a molecular repertoire size *N*_G_ = 100, as used in most of our simulations. It turns out that for an assembly size *N* = 36 (a small micelle), the total number of possible compositional combinations is 7.7 × 10^32^ (based on the compositional information formula of figure 4). This means that at a given time point on early earth *all* the possible compositions defined by the above parameters may well have been present. This, in turn, implies that somewhere on planet earth even the strongest possible mutually catalytic network with these parameters (likely a composome) would be present, even before the onset of GARD attractor dynamics. These probability computations are not in the realm of the Bertrand paradox [247], as the method of random selection is very well specified by a simple combinatorial formula, in which all combinations can be numerically assayed. The above argument cannot be taken simply as evidence that life is highly probable. But this depiction provides quantitative routes for addressing the question of life's probability. This might begin to resolve a long-term dispute, eloquently presented by Shapiro [12, p. 117]. He first quotes Monod [248]: ‘The universe was not pregnant with life, … our number came up in the Monte Carlo game’. Shapiro then supportively quotes de Duve [249, p. 112]: ‘life arose through the succession of an enormous number of small steps, each … had a high probability of happening. This assumption simply amounts to a rejection of improbabilities so incommensurably high that they can only be called miracles'. The GARD planetary scenario shows that on a large planet very small probabilities may materialize, and can in principle help translate this statement into tangible numbers. This could constitute a step towards proving Shapiro and de Duve right regarding whether the universe is pregnant with life [250].

An enlightening relevant clause is put forth by Davies [251, p. 6]: ‘It makes sense to try to explain life's origin only if it resulted from processes of moderately high probability, so that we can reasonably expect to give an account in terms of known science. It then follows from simple statistics that there will have been a large ensemble of systems proceeding down the pathway toward life, and no obvious reason why only one member successfully completed the journey. Ideally then, there should be a parameter, or more probably a set of parameters, to quantify progress towards life’.

The idea of decillion parallel assemblies, some of which being replicators right at their accretion, is intriguing. Could it be that (very primitive) reproduction, and possibly evolution had materialized on earth very near to the first appearance of aqueous organics [166]? While this is not an impossibility, it is likely very optimistic. The very early GARD-style potential replicators must have been too rare, too feeble to survive for long, and with slow and inaccurate reproduction capacity. Perhaps the right picture is that of a vast collection of membrane structures (vesicles, micelles, reticuli) surrounded by hydrophobic oil droplets as well as amphiphiles and soluble monomers. The membranes were undergoing incessant accretion and decomposition, fission, budding and fusion dynamics. Ever so slight compositional homeostasis emerged here and there, based on the GARD principles. The assemblies involved created ‘colonies’ of (initially compositionally rather remote) copies, thin clouds in compositional space. These got more focused as better composomes were reached via catalysed monomer exchanges. And as reproduction improved, better capacity to evolve would show up by the paths described in §§ 7 and 11.1. This might have been the somewhat fuzzily defined point at which life (by NASA's definition) could have emerged.

There is one last point that needs to be mentioned. Nowak [236, p. 14924] asks a fundamental question, pertinent to planet-scale dynamics: ‘When do chemical kinetics become evolutionary dynamics?’ He points out that ‘Evolution needs populations of information carriers’ (usually considered as) ‘derivative of replication’. Using a bit string computer model of growing polymers, he reaches the conclusion that ‘Replication is not a prerequisite for selection, but instead, there can be selection for replication’. This is fully analogous to what Higgs calls ‘selection without replication’ [71, p. 225]. Nowak calls ‘such a system prelife and the associated dynamics prevolution’. His simulations, as described, ‘can define a prebiotic chemistry that can produce any binary string and thereby generate, in principle, unlimited information and diversity’ needed for selection and evolutionary capacities. The decillion planetary assemblies scenario conforms precisely to Nowak's description of prolife and prevolution and Higgs' depiction of selection without replication. The GARD planetary scenario predicts the random formation of an astronomical number of compositional assemblies, a huge majority of which are non-replicating, hence belonging to prelife and prevolution. But the planetary scale statistics plus GARD dynamics ascertain a relatively high probability of transition to replication and evolution.

## 14. Eventual evidence

Can any model for the origin of life be experimentally tested? It is unanimously agreed life's origin has been a very long chain of spontaneous chemical reactions, without any external intervention of the kind exerted in many relevant laboratory experiments. Further, as life likely arose over very long periods of time and in huge volumes, one might have to perform experiments involving ‘test-tubes’ of cubic kilometres and lasting millions of years. An alternative would be explorations of extrasolar planets seeking instances of just-emerging life. Owing to the lack of immediate realism in both such approaches, the community is forced to scrutinize miniscule fragments of the origins scene (see detailed discussion in [59], §11.3.1).

But are we looking at the correct fragments? It turns out that practically all the relevant experimental studies explore the abiotic synthesis and mutual interactions of individual chemical entities, almost always similar to those found in present-day living cells (see §2). Curiously, when attention is turned to systems views, many fewer experiments are performed. The reason is that such systems are often too complex to be fathomed experimentally in standard laboratory settings. This creates a chasm in the field between the experimentalists and the computer simulation scientists, with the former usually considering the simulation results as having little practical significance. Such an attitude seems oblivious to the fact that large-scale complex phenomena, such as the weather, planet accretion/collision and galaxy formation are studied almost exclusively by computer simulations [252]. It is quite possible that in the last account, life's origin is yet another such large-scale realm. GARD has been formulated with such a possibility in mind.

In the more specific framework of the GARD model, a question is often asked: can one make vesicles composed of 100 different amphiphile types and hope to see homeostatic growth as predicted by the simulations? The answer is no, not because this is an impossibility, but because such dynamic behaviour is very slow, inaccurate and combinatorically very rare. However, the ray of hope is that if computer simulations are provided free reign, as discussed in the next section, computer-based guidance would help to intelligently plan the necessary large-scale laboratory experiments.

### 14.1. Future computing evidence

Future hope could be in the realm of high power computing. It is relevant to quote Dyson's thoughts on this topic [8, p. 77]: ‘In population biology … the computer is a source of experimental data at least as important as field observation. Computer simulations of population dynamics are indispensable for the planning … and for the interpretation of results … Every serious program of research in population biology includes computer simulations as a matter of course. Because the origin of life is a problem in the population biology of molecules, computer simulations are essential here too’.

Indeed, early molecular dynamics simulations of lipid bilayers provided insight important for kinetic models such as GARD. This is exemplified by demonstrations by Pohorille that the membrane–water interface forms an environment suitable for heterogeneous catalysis [253] and that small molecule entry dynamics should take into account specific interactions at three different depths, lipid headgroup layer, rigid hydrocarbon chain layer and the deep fluid hydrocarbon in the centre of the bilayer [254]. Numerous other studies have used molecular dynamics to probe lipid and membrane behaviour as reviewed [255]. Of particular relevance to GARD are molecular dynamics studies that provide detailed information on bilayer entry and exit of individual lipid molecules, including inference on thermodynamic and kinetic parameters [140,255,256].

The last few years have seen tremendous progress in the use of molecular dynamics in biological chemistry [257], including in studies of life's origin [253,258]. It has become possible to use this computer simulation method to predict the folding of small proteins [259], a feat considered next to impossible 10 years ago. Likewise, ligand protein interactions are now being similarly simulated, including the forward kinetic constant for ligand binding [260], and so are enzyme-substrate catalytic interactions [260]. Similar simulations are performed to predict the mode of self-assembly of membrane [261], including the incorporation of proteins into the bilayer [262]. With appropriate near-term improvements, it may become possible to simulate the mutual interactions of various lipids in a small assembly (e.g. micelle), and perform computerized molecular dynamics screens for mutual interactions within diverse amphiphile combinations. This could help elucidate mutual rate enhancements, and be used to select appropriate specific amphiphile compositions for experimental studies of GARD dynamics.

Fifteen years ago, we published a paper entitled ‘Prospects of a computational origin of life endeavor’ [263]. It made the claim that ‘As computational tools for the reconstruction of molecular interactions improve rapidly, it may soon become possible to perform adequate computer-based simulations of prebiotic evolution’ [263, p. 181]. This foresight is not yet fulfilled, but it is clearly visible on the horizon. A fascinating paper by Borhani and Shaw discusses the future of molecular dynamics simulations between now and the year 2037 [264]. The paper uses rigorous reasoning, based on Moore's Law for the increase of computing power along time, and analyses in detail the novel methodologies expected to emerge. One of the mottos of this paper is that molecular dynamics analyses of biomolecular recognition will ‘achieve an accuracy equal to or better than typical experimental binding or activity assays’ [264, p. 21]. An important forecast is that molecular dynamics will be able to analyse structures up to a 1-µm scale in acceptable simulation times. This means complete prediction of the folding of a large protein, the activity of large protein complexes including those participating in DNA replication and transcription, protein–protein and protein–ligand interaction and enzyme kinetics, including free energy calculations. At the far end, the authors even mention simulations of entire organelles and whole bacteria.

Such advanced technology should allow full-fledged computer ‘experiments’ for origin of life scenarios, including the GARD and M-GARD models. Molecular dynamics-based GARD is envisaged, among other directions, as involving network kinetics simulations, in which the rate constants are derived from molecular dynamics, as reported for individual reactions [260,265,266], as well as for reaction networks [267]. In this respect, the substantial body of GARD kinetic simulations already performed form a basis for future, more broadly disposed GARD molecular dynamics.

Another relevant article is a recently published 25-year forecast for a related methodology, computational chemistry [268]. Notably, the above computational forecasts do not take into account the incredible revolution that could transpire with quantum computing, which has realistic prospects for certain chemistry-related computations [269].

It is important to clarify what types of origin of life models are amenable to molecular dynamics and computational chemistry analyses. Such models should be based on real molecules, e.g. peptides or lipids, not bit strings or imaginary replicators (the latter should be specified in kinetic detail as described [59]). The models should also allow breakdown to specific questions about chemical reactions. For example, what is the degree of catalysis exerted by peptide X at a given aqueous concentration on amide bond formation between derivatized peptides Y and Z at their own specified concentrations (Kauffman's model)? Or, what will be the combined rate enhancement of 20 specified lipid types at given molar fractions in a micelle on the entry of one of those lipid types with a defined extraneous concentration (GARD model)? In a summary, models that are kinetically specified, with molecular identities and atomic structures, concentrations, binding constants, rate constants and equations describing time dependences are better compatible with molecular dynamics.

As mentioned, life's origin may better be regarded and analysed as a large-scale complex phenomenon. However, these methods are unlikely to reconstruct the exact history, but have a fair chance to shed light on some important underlying principles. What distinguishes life's origin from some of the other fields mentioned above is that life has much higher complexity at the molecular level. Leveraging ideas and methodologies from other simulation disciplines, while keeping a mind open regarding crucial differences, should benefit future attempts to solve the origin of life riddle.

## 15. Conclusion

Life is defined as what replicates and evolves, but its emergence paths are still widely disputed. Steps needed to break the stalemate have been outlined by Walker *et al.* [270, p. 6]: ‘This necessitates a re-conceptualization of the origins of life, removing the imposed hard boundary between non-life and life, and recognizing there may exist physical processes that we do not yet understand … One candidate is the physics of information’. Accordingly, we describe here a physico-chemical line of attack that defines the life/non-life boundary at the molecular level, and explores the use of the unorthodox platform of compositional information.

Our GARD approach constitutes an extension of a well-documented scenario of mutually catalytic networks. One of crucial modifications is that the model is amphiphile-based, affording spontaneous accretion, flexibility in the involvement of molecule types (opportunism), facile mutual reactions, random access due to fluidity and a propensity for fission. Another unique property is that GARD shows emerging composomes, privileged compositions whose replication (or reproduction) is an emergent outcome of the kinetics and not arbitrarily assumed. Through GARD, we strive to carefully assess the validity of the autocatalytic set school of thought, and seek evidence for its legitimacy as a *bona fide* scenario for life's origin.

Compositional reproduction is unfamiliar to the field, but is highly prevalent in life today. In present-day cells, it strongly depends on sequence copying and translation, leading to the adamant dogma that without templating polymers life could not have emerged. An important message stemming from GARD scrutiny is that compositional information transfer *could* predate polymer sequence copying. To make a convincing case, we devote special efforts to exploring the criteria that justify describing an autocatalytic set as a replicator. This includes the assertion that merely having the production of each network member assisted by mutual catalysis is insufficient, and that homeostatic growth of the set has also to be demonstrated.

We address the doubts regarding whether mutually catalytic networks may evolve. We show that the lability of single compositional mutations does not stand in the way, because compositional information stability is assured by the attractor dynamics of entire composomes. We show how environmental chemical changes induce transitions from one composomal state to another, including preliminary evidence for open-ended compositional evolution.

Related to a paucity of basic GARD in lacking catalysed covalent chemistry, a likely prerequisite for evolvability, we present a new model version, M-GARD, which includes covalent modifications governed by non-enzymatic lipid catalysts. We provide extensive evidence for the realism of such catalytic capacities, and mention studies showing that composomes dynamics includes the products of covalent bonding, e.g. dimers. This upgraded model thus helps solve a long-standing problem of the metabolism first scenario: the need to replicate not only the metabolites but also the catalysts that afford their production.

Regarding evidence for our model's validity, we point out the insurmountable hurdles for comprehensive experimental verification, but highlight a first published experimental instance of a simple lipid-based GARD network [193]. In this vein, we point to published educated predictions that, within a decade, it will become possible to fully simulate M-GARD by immensely accelerated molecular dynamics. This would fully verify that relatively complex GARD protocells might portray full homeostasis-based replication/reproduction, including both bilayer and lumenal contents.

The GARD model thus offers vistas, which are not readily available to many other origin scenes. The widely accepted view, exemplified in a paper by De Duve [217] (figure 16*a*), calls for life's origin in three parallel threads, deriving from three classes of abiotically formed chemical compounds: fatty acids that form membranes, amino acids that form proteins and nucleotides that form replicating RNA. The published figure (figure 16*a*) does not reveal how the three threads join to form a full-fledged protocell.

GARD and M-GARD, on the other hand (figure 16*b*), call for early thread-joining in a lipid protocell. Opportunistic amphiphile headgroups may encompass amino acids and peptides, RNA nitrogen bases and oligonucleotides capable of base pairing, cofactors, metal chelators, thiols, oligo-phosphates and numerous other compounds that do not appear in life today. By the M-GARD rulebook, such diversified compounds in the membrane and vesicular lumen constitute a grand mutually catalytic network, involving both non-covalent and covalent reactions. This compositionally replicating protocell is equipped to launch a long evolutionary journey, via many hard-to-fathom intermediates, including unorthodox chemistries such as non-canonical amino acids and nucleotide bases as well as unconventional polymers such as polyesters [271] and peptide nucleic acids [272], all the way to the LUCA.

GARD's systems view goes beyond integrating chemical threads. It shows conformity to the continuity principle that puts the burden of proof on any theory that requires discontinuities [104]. Unlike RNA-first, GARD reaches a network-based multi-molecular replicator via small ‘distillation’ steps, followed by gradual improvements. The systems-prone GARD also portrays many of the properties of living systems, which cannot be readily identified in ‘naked’ replicating RNA.

The adequacy of GARD to serve as a model for pre-RNA life is also demonstrated by the observed thermodynamic and kinetic traits that befit a precursor to present-day living cells. This includes attractor-like transitions from random assemblies to self-organized composomes, which involve a negative entropy change. These facets, along with permanently being away from equilibrium, exchanging matter and energy with its environment and being able to amplify small fluctuations, establish GARD composomes as dissipative systems, hallmarks of life.

Finally, we consider GARD composome's role in an origin of life scene at planetary time and volume scales. We computed that a fraction of the Hadean ocean surface layer could easily fit a decillion (10^33^) GARD assemblies. In turn, GARD combinatorial computations suggest that, for acceptable assembly size and repertoire count, approximately 10^33^ different compositions are possible. Thus, every possible composition will have been materialized, including some of the most effective mutually catalytic networks. This profoundly implies that effective composome replicators could have emerged soon after the terrestrial oceans formed 4 billion years ago. In parallel, we indicate that it took most of the next 0.5 billion years for the first network replicator to evolve towards the first fossil-visible protocellular entity at 3.5 billion years ago. Such planetary insights could assist the search for early life on other planets.

## Endnotes

1 There is a prevalent sentiment for the requirement to distinguish between ‘self-replication’ and ‘self-reproduction’, as exemplified in [7]: ‘The distinction between reproduction and replication is a relevant one to make here in order to clarify the different types of processes to which we will be referring, as well as their final outcomes. We follow Dyson's insight [8] on this point: the term 'replication' will convey any reliable copying process, taking place at the molecular level, that gives as a result new molecules that conserve the specific sequence of a pre-existing one (commonly called the template). In turn, the term ‘reproduction’ will be used as a more general concept that involves the spatial multiplication or division of a whole system and is not necessarily reliable, in a statistically meaningful sense, as far as the production of identical copies is concerned’. The two comparative criteria offered in the quote above are the number of molecules involved and the exactitude of copy-making. Whenever possible we follow this distinction. However, we note that requiring a special term for multi-molecular entities might defy a central goal of origin of life research, namely asking unbiasedly what were the first entities capable of copying themselves, irrespective of size, fidelity and complexity. This is reflected in NASA's definition of life. Regarding fidelity, we note that isolated early templating polymers may have possessed similarly low copying precision when compared to early mutually catalytic networks.

2 In this paper, we use ‘composome’ to generally indicate the dynamic entity which is a compositionally replicating assembly. The term ‘compotype’ is used in specific instances in which clusters of composomes are specifically addressed.

## Data accessibility

Published data are accessible as indicated in the respective quoted publications. Unpublished data are available upon request from the corresponding author.
